# The influence of new information that contradicts common knowledge about earthquake preparedness in Israel: A mixed methods experiment study

**DOI:** 10.1371/journal.pone.0250127

**Published:** 2021-04-14

**Authors:** Anat Gesser-Edelsburg, Mina Zemach, Ricky Cohen, Talya Miron-Shatz, Maya Negev, Gustavo S. Mesch

**Affiliations:** 1 The Health and Risk Communication Research Center, University of Haifa, Haifa, Israel; 2 School of Public Health, University of Haifa, Haifa, Israel; 3 Midgam Consulting and Research, Bnei Brak, Israel; 4 Center for Medical Decision Making, Faculty of Business Administration, Ono Academic College, Kiryat Ono, Israel; 5 Department of Sociology, University of Haifa, Haifa, Israel; Neijiang Normal University, CHINA

## Abstract

**Background:**

A major earthquake in Israel is inevitable. Individual risk perceptions and preparedness can mitigate harm and save lives. The gap between the public’s concerns and those of experts is reflected in their differential perceptions regarding the components that influence the occurrence of an earthquake in Israel. Whereas the public believes that geographic location is the critical variable, the experts note additional variables that need to be considered. Common knowledge regarding the risks of earthquake occurrence in Israel is based on a distinction between high and low-risk areas, such that the closer a residential area is to the Great Rift Valley, the higher the risk that an earthquake will occur.

**Objectives:**

To examine the variables affecting public preparedness in Israel (effective communication agent (communicator), high and low earthquake risk areas) and the degree to which experts’ knowledge contradicts respondents’ common knowledge.

**Methods:**

The study used a mixed-methods approach combining qualitative and quantitative research. The first stage included in-depth interviews with earthquake experts (n = 19). The second stage consisted of an experiment conducted among a representative sample of the public (n = 834).

**Results:**

Most people believe that geographical location constitutes the main risk factor for earthquakes in Israel. Yet experts claim that additional variables affect earthquake intensity and damage: building strength, earthquake magnitude, distance from earthquake epicenter, soil type, and interaction between these four. The study found that knowledge of expert information affects public willingness to prepare. The direction of this influence depends on participants’ risk perceptions regarding residential area and on degree of consistency with common knowledge. In low-risk areas, added knowledge increased willingness to prepare whereas in high-risk areas this knowledge decreased willingness.

**Conclusion:**

To turn expert information into common knowledge and to increase earthquake preparedness, the authorities must educate the public to generate a new public preparedness norm.

## Introduction

### Variables affecting willingness to prepare for an earthquake

Most studies on public earthquake preparedness sought to examine factors predicting which variables play a decisive role in people’s attitudes and behavior [1–5]. These studies revealed a great deal of controversy surrounding the variables associated with preparedness and the influence they exert.

Many studies over the past 45 years associated respondents’ adoption of seismic hazard precautions with risk perceptions, demographic characteristics, personal experience, social influence, and other variables. In a systematic literature review, Wachinger et al. [6] claim that cultural and individual factors such as media coverage, age, gender, education, income, social status, and others do not play such an important role as primary predictors of preparedness but rather act as mediators or amplifiers of the main causal connections between experience, trust, perception, and preparedness to take protective actions. Some studies indicate that disaster preparedness is positively associated with risk perception [7, 8]. Yet others show that there is not necessarily a direct link or that only a weak link exists between awareness, perceived risk and desired preparations or behavioral responses [9–12].

Current research indicates that trust in government is negatively associated with risk perception and preparedness. As people place more trust in the government, their perceived risks of an earthquake occurring are lower and accordingly they take fewer protective measures [13–15].

Han, Lu et al. (2017) explained this negative correlation between trust and preparedness by noting that the subjective experience of effective government support seems to have a side effect of diminished individual resilience. This unexpected effect should be taken into consideration in disaster and emergency management [13]. For example, a survey of 501 households in a Tibetan area of China that was hit by the 2010 Yushu earthquake examined trust in government and individuals’ risk perception, as well as their perceived earthquake preparedness. The results indicated that people with higher degrees of trust in government perceive lesser consequences of potential earthquakes and tend to prepare less [13]. On the other hand, a positive association emerged between previous earthquake experience among respondents or their significant others [16] and hazard adjustment [16, 17]. For example, McClure et al. [17] examined preparedness and judgments of earthquake risk in three New Zealand cities after the 2011 Christchurch earthquake. The research examined the association between participant’s city of residence and risk assessments before (recall) and after the earthquakes, participants’ attributions for their risk judgments and for (not) preparing, and earthquake damage for Christchurch participants. The findings suggest that prior expectancies and disaster experiences affected earthquake risk judgments and preparation both inside and outside the affected area.

In places in the world such as Israel, where no major earthquakes have yet occurred, individual experience cannot be used determine how people assess the risk of an earthquake. Hence these assessments must be established by the opinions of experts on the subject.

### Public vs. expert risk perceptions and realism

The risk perception literature points to the importance of understanding differences between public opinion [18] and expert opinion. Several studies in the seismic risk perception literature examined the correlation between public risk perception and *material risk*, defined as “risk conceptualized scientifically as a probabilistic statement regarding degree of future hazard and exposure” [12]. Some studies found a correlation between material seismic risk and public risk perception, such that the US public was more optimistic than the experts regarding the probability of an earthquake [1, 3]. In Europe, a cross-national study found a correlation between objective earthquake risk (based on hazard occurrence data) and the public’s seismic risk perception. The experts relied on advanced technologies that have improved the ability to predict dangerous phenomena, although earthquakes are still impossible to predict with complete accuracy [1, 3].

Some studies have shown that there is no clear-cut division between expert knowledge and public knowledge. An analytical division of this nature can lead to inaccuracies, since the public’s thinking is often complex rather than one-dimensional. For example, Slovic et al. [19] explored the association between the analytical and emotional aspects of risk perception, and specifically the association between analytical risk analysis and experience-based risk perception. The "analytical system" model addresses a person’s ability to analyze rules and norms and calculate risks and opportunities, whereas the "experiential system‴ model is intuitive, quick, automatic and partially subconscious. The findings indicate that the public is not irrational. Rather, the public evaluates risks based on criteria and values that are not necessarily obvious to experts but that are relevant to the everyday lives of some members of the public [20].

According to Sandman [21], risk perception consists of hazard level (risk estimation) and outrage level (the emotions stimulated by the risk). If the public’s hazard and outrage levels are similar to those of the experts, the risk can be communicated very effectively, but if these levels differ between the two groups, controversy is liable to ensue. In order to communicate a risk successfully, organizations should consider the public’s feelings about the risk and make efforts to minimize the gap between experts’ risk estimations and the public’s risk perceptions.

In comparing experts’ perceptions of different risks (e.g., natural disasters, politics) to those of the public, Sjöberg [22] found differences between the experts and the public regarding risk evaluation in different situations. This discrepancy points to the public’s mistrust of expert assessments. This mistrust stems mainly from misunderstanding the risk, causing the public to be skeptical of experts’ predictions. Various components can explain this variance between public and expert risk perceptions. One of these components is the differential approach to what Sjöberg calls "realism": The public may in fact be misinformed and the experts may be making realistic risk assessments.

The question, then, is how to reduce the gap between expert and public assessment of earthquakes to ensure that the public’s risk perception is realistic. One of the strategies in persuasion communication is to provide new expert information or information that is only partly familiar to the public in order to raise the public’s consciousness and influence its risk perception. Yet different theories indicate that in order to avoid creating a potential "boomerang effect" [23, 24] that might lead the public to reject the new information, in this case about earthquake preparedness, experts must prepare the public for this new information.

According to the inoculation theory, the public needs to be “inoculated” in advance to prevent it from rejecting the new message/information. This theory posits that an attitude or belief can be safeguarded against persuasion or influence in much the same way the body can be protected against disease, i.e., through pre-exposure to weakened versions of a stronger future threat [25, 26]. The theory uses medical inoculation as an analogy and applies it to attitudes (or beliefs) rather than to disease. This approach has major potential for building public resilience ("immunity") against misinformation and fake news, for example by attacking science denial, risky health behaviors, and emotionally manipulative marketing. McGuire [24] described how attitudes change, and more specifically how to keep existing attitudes and beliefs consistent in the face of attempts to change them.

Authorities have a role to play in conveying the information available to experts in a way that is understandable and accessible to the public. In order to create resilience and good assessments among the public, research must also consider countries such as the State of Israel, where people do not have personal experience with earthquakes despite the risk of an earthquake occurring.

### Israel and earthquakes

Israel is located along the Great Rift Valley, a region that includes Lebanon and the Sinai Peninsula and is marked by major seismic activity. The first devastating earthquake to hit the area of the current State of Israel for which seismometric data are available took place in 1927. This earthquake had serious consequences: 285 dead, 940 wounded and extensive damage to buildings. The earthquake severely affected the cities of Jerusalem, Jericho, Ramla, Lod, Tiberias and Nablus. The seismic magnitude of the earthquake was assessed on the MSK scale in 133 localities. A maximum seismic magnitude of IX on the MSK scale was measured along the Jordan River, with the epicenter in the northern Dead Sea. The previous severe earthquake to hit the area of the current State of Israel was in 1837, completely destroying the city of Safed [27, 28].

On November 22, 1995, the strongest earthquake ever measured in the area occurred in the Gulf of Eilat. Its magnitude was 7.1 on the Richter scale, and its epicenter was about 100 km south of the cities of Eilat and Aqaba. Due to the relatively large distance from populated areas, this earthquake did not cause severe damage or many deaths.

Among the main risks to human health in the event of an earthquake are the partial or complete collapse of buildings, bridges and roads as well as nonstructural failures. According to the National Steering Committee for Earthquake Preparedness, a major earthquake in Israel may result in 7,000 casualties, 8,600 citizens with severe or moderate injuries, 9,500 people trapped in buildings, and 170,000 displaced persons [29].

The 2018 Israel State Comptroller’s report [30] found that the State of Israel is not prepared for an earthquake because no long-term measures have been taken so far. Such measures include warning systems, strengthening the infrastructure of public and residential buildings and establishing a system of compensatory insurance. Moreover, the Steering Committee is a government committee that acts as a professional body for coordinating and directing the government’s activities in the field of earthquake preparedness, but it has no operational powers at all. Indeed, responsibility for implementation has been given to the National Emergency Management Authority, a body that deals with home front preparedness. The report sharply criticizes the fact that to date the Steering Committee’s detailed plans are a recommendation only and calls for ensuring the status and independence of this committee.

On average, an earthquake with a magnitude of six or higher on the Richter scale hits the region about once every eighty years [31], though several centuries have gone by without a major earthquake. Past earthquakes caused grave damage to life and property. In modern times, Israel has been hit by a number of low magnitude earthquakes [28, 32]. Experts agree that a major earthquake will hit Israel in the future, though they cannot predict the precise time, place or magnitude [28, 33]. In 1980, Israel adopted a new seismic building code according to which buildings three stories or higher built prior to 1980 require seismic retrofitting. This code has been implemented mainly in the affluent parts of Israel [34].

The last major survey of public perceptions of earthquakes in Israel was conducted in 2015 and found no significant differences in level of earthquake preparedness between residents of high-risk areas (close to the Great Rift Valley) and areas not defined as high-risk. In addition, the Israeli public’s assessment of earthquake risk is not high, and this low assessment is apparently associated with a relatively low level of public preparedness [35].

Another Israeli study conducted in 2018 by Shapira et al. [16] examined behavior strategies among residents living in high-risk areas for earthquakes. The study found that residents’ preparedness level was positively associated with their education level, income, and previous emergency experience and negatively associated with their risk perceptions. In other words, precisely those who perceived the risk of earthquakes as lower were the ones who prepared more.

It is hard to find people who personally survived a major earthquake in Israel. Therefore, the critical variable of “personal experience” cannot be measured in Israeli studies. This may be why the survey by Shapira et al. asked respondents about their previous emergency experience in general and may also explain the fact that the Israeli public’s level of concern regarding earthquakes is low [16]. On the other hand, experts in Israel point to the high likelihood that a powerful earthquake will occur and that the country needs to prepare accordingly, as underscored by the following quotation from the 2018 State Comptroller Report: “Professionals believe that a strong earthquake in Israel is a certainty. There is no choice but to prepare for one” [33].

Israeli experts and the Israeli public differ in their predictions regarding whether an earthquake will occur in Israel. The public receives most of its information from the media, which emphasizes the variable of geographic location, namely, the division of the country into higher and lower risk areas [36, 37]. The experts, in contrast, point to additional variables that need to be taken into account: building strength, earthquake magnitude, distance from earthquake epicenter, soil type, and the interaction between these four factors.

This study seeks to examine the variables that influence preparedness among the Israel public, and in particular the impact of expert knowledge. The study is unique in that we used an experiment to examine the study participants according to variables that have not been tested in other studies. In particular, we examined the impact of new information about the risk factors and damage caused by earthquakes possessed by experts but not part of the common knowledge of the experimental participants.

### Research goals

The goal of this research is to identify the influence of the following variables and to examine how they interact while communicating information to the public to motivate it to prepare for an earthquake:

- Most effective communication agent (communicator): The experiment tested three relevant options to determine who is the most effective communicator of earthquake information (Homefront Command spokesperson, mayor of respondent’s hometown, a geologist).
- Transmission of new information that influences people’s chances of being harmed by an earthquake: Can the transmission of new information change common knowledge about the likelihood of an earthquake and the public’s intention to prepare for one, and under what conditions?
- Area of residence: Areas that according to common knowledge are considered high-risk areas for an earthquake versus areas where according to common knowledge the risk of an earthquake is considered relatively low.

## Materials and methods

### Research design

The study used a mixed-methods approach that combined qualitative and quantitative research. The qualitative section entailed in-depth interviews conducted with experts on earthquakes. The quantitative section consisted of an experiment to examine the variables that was conducted among a representative sample of the public.

The combined array of quantitative and qualitative research methods followed a sequential design [38, 39]. In the first stage we conducted interviews with experts in order to identify the factors that lead up to an earthquake and determine its magnitude and the odds of being injured. The second stage consisted of a before-after experiment. In the “before” stage we examined people’s perceptions regarding the above issues and their reported level of earthquake preparedness. In the “after” stage we examined the correlations among a number of variables determining compliance with earthquake preparation recommendations.

The interviews with the experts were intended as an exploratory design system. Namely, this qualitative research was intended to enable us to examine and describe the reasons earthquakes occur in order to design the contents of the information given to the experimental participants. The before data of the experiment also enabled us to estimate the association between the information emerging from the expert interviews and the experimental participants’ preparedness variables [40].

### Research population

We used purposeful sampling to select the interviewees in the qualitative stage [41, 42]. We interviewed experts from a variety of fields to discover various causes for earthquakes in the context of the research goals. Nineteen experts were interviewed: geology and seismology experts (past and present employees of the Geological Institute), academics and government officials, officeholders in organizations responsible for emergency preparedness (Homefront Command, National Emergency Authority, Earthquake Preparedness Steering Committee).

In the experimental study, we classified all the localities in Israel into two groups according to participants’ area of residence: areas that according to common knowledge are considered at high-risk for an earthquake (geographically closer to the Great Rift Valley) versus areas that according to common knowledge are considered to be at relatively low-risk for an earthquake.

We then used the strata sampling method to extract a sample for each of these two categories. The criteria for stratification definition were nationality and sector (immigrants from the Former Soviet Union from the 1990s and onward, Jews, others, Arabs) and gender. Each stratum was represented in the sample relative to its proportion in the Israeli adult population. A total of 834 respondents participated in the survey: 368 from high-risk areas and 466 from low-risk areas.

### Research process

Qualitative research: The personal face-to-face interviews with the experts were conducted in March 2019 by two research assistants who had been trained in qualitative interviewing. Each interview lasted between 40 minutes and an hour. All the interviews were recorded and transcribed by the research assistants.

Quantitative research: The participants were sampled from iPanel, an Israeli internet panel. They were interviewed online in July 2019. The experimental design (S1 and S2 Appendices) began by testing the participants before the experimental manipulation (hereinafter: before). Participants then underwent the experimental manipulation. They were given recommendations for correct earthquake preparation followed by repeated test of some of the dependent variables immediately after the experimental manipulation.

**Fig 1:**
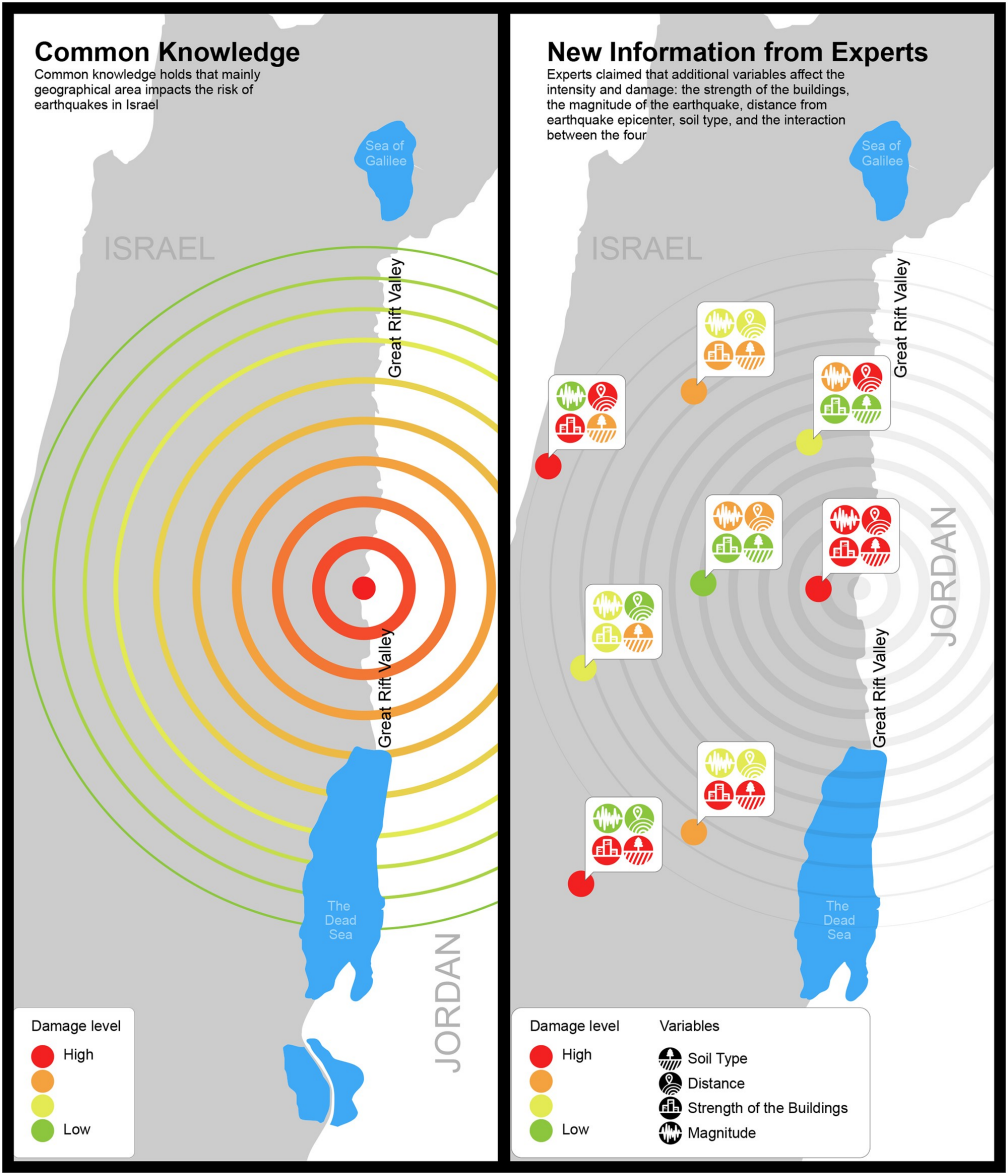
Common knowledge versus new information from experts regarding the earthquake factors. According to common knowledge, geographical area is the main factor affecting the risk of earthquakes in Israel, whereas new information from experts adds variables that affect earthquake intensity and damage: building strength, earthquake magnitude, distance from the earthquake epicenter, soil type, and the interaction between these four variables.

The independent variables were as follows: communicator identity (three different persona), type of information communicated (common sense vs. common sense plus expert knowledge), and geographical area (corresponding with perceived earthquake risk for the area). Each participant was exposed to one communicator in the experiment: the Homefront Command spokesperson, the mayor of the respondent’s hometown, or a geologist. Each participant received one form of information from the communicator: either information that was common knowledge (i.e., that the decisive factor regarding earthquakes is geographical area) or “new” information (i.e., information based on our interviews with the experts who claimed that factors in addition to geographical area affect earthquake occurrence and intensity). Fig 1 depicts common knowledge versus new information from experts regarding the earthquake factors.

The texts for the two groups were equal in length. In the common knowledge condition, the following text was provided:

*Hello*. *I am*… *I would like to speak to you about preparedness for an earthquake in Israel*. *In the past*, *destructive earthquakes have occurred in our area*, *and the occurrence of another one is only a matter of time*. *There is definitely going to be an earthquake but we do not know where or when*. *Over the years*, *numerous geologists in Israel and worldwide have been trying to develop tools to predict earthquakes*. *These geologists are also trying to calculate the chances for an earthquake by specific area and to predict its intensity*. *As we have already said*, *despite intensive efforts over many years by the world’s best geologists*, *we cannot yet predict when or where an earthquake will occur*, *or how strong it will be*.

In the “new information” condition, the following text was provided:

*Hello*. *I am*… *I would like to speak to you about preparedness for an earthquake in Israel*. *In the past*, *destructive earthquakes have occurred in our area*, *and the occurrence of another one is only a matter of time*. *There is definitely going to be an earthquake*, *but we do not know where or when*. *The chances of being injured and the level of harm depend on a combination of the following factors*: *the strength of the building you live in; whether it was built to meet emergency standards; the magnitude (strength of the earthquake); distance from the epicenter; and kind of soil*. *We must emphasize that contrary to conventional thinking*, *the level of damage from earthquakes does not depend on geographical area alone but on a combination of the four factors cited above*.

Finally, the variable of area of residence was binary: low-risk versus high-risk, i.e., areas that according to common knowledge are at high-risk for an earthquake (geographically closer to the Great Rift Valley) versus areas that according to common knowledge are at relatively low-risk of an earthquake.

The combination of the three independent variables generated the following 12-cell model (Table 1). For the purpose of the experiment, participants were randomly divided into 12 groups. Examination of participants’ sociodemographic profiles indicated no variance between the 12 groups.

**Table 1:** Combination of the three independent variables for the experimental groups (n = 834).

Area of residence	Communicator and information transmission by parties responsible for controlling earthquake damage						
	Homefront Command spokesperson		Geologist		Mayor		Total
							n (%)
	New information presented	Common knowledge information	New information presented	Common knowledge information	New information presented	Common knowledge information	
	n (%)	n (%)	n (%)	n (%)	n (%)	n (%)	
**High-risk**	70 (8.4)	62 (7.4)	59 (7.1)	55 (6.6)	61 (7.1)	61 (7.3)	368 (44.1)
**Low-risk**	72 (8.6)	76 (9.1)	78 (9.4)	82 (9.8)	81 (9.7)	77 (9.2)	466 (55.9)
**Total**	142 (17.0)	138 (16.5)	137 (16.4)	137 (16.4)	142 (17.0)	138 (16.5)	834 (100)

### Research tools

For the qualitative research with the experts, we designed a semi-structured interview protocol that covered the following topics: elements that influence earthquake occurrence; likelihood of an earthquake in at-risk areas (e.g., What do you think are the high-risk and low-risk areas for earthquakes? What do you think are the chances of a major earthquake in high-risk areas in Israel? From your professional experience, have you come across different risk assessments among local and international seismic experts?); uncertainty and behavioral elements (e.g., How do decision-makers deal with the uncertainty surrounding earthquake prediction?); public guidelines—preparation and behavior (e.g., what do you think are the public’s barriers to earthquake preparation?); risk communication and earthquake preparation (e.g., If you were the spokesperson today, what are the most important messages you would convey the public?).

For the experiment, we designed a questionnaire that included sociodemographic characteristics and that examined the following variables: risk perception of chances of an earthquake, level of concern about being harmed by an earthquake, socio-cognitive characteristics (internal or external locus of control, self-efficacy), awareness of danger of earthquakes in the interviewee’s area (considered as mediator variables). The dependent variables were the interviewee’s actual preparation, as determined by a) a general question, b) questions about complying with the guidelines on the Homefront Command website (e.g., seismic retrofitting of buildings three stories or higher built before 1980 to bring them up to the 1980 seismic building standard, avoiding hanging pictures and other objects above beds and securing items hanging on the walls, stockpiling water, food, medicine, flashlights and so on), and c) questions about subjective preparedness norms. The dependent variables determined by the actual preparation scale were measured on a 4-point scale: (1) I have already performed the recommended action, (2) I intend to perform the recommended action in the near future, (3) I intend to perform the recommended action in the distant future, and (4) I do not intend to perform the recommended action.

After participants were exposed to the experts’ information, they were again asked about the dependent variable, i.e.. their preparation intentions. To examine the success of the manipulation involving transmitting new information, participants were asked to indicate what factors are responsible for earthquake occurrence and intensity and to assess their chances of being harmed.

### Questionnaire reliability and validity

Before running the experiment, we ran a pilot with 104 participants to test the split-half reliability [43, 44]. The pilot found a correlation coefficient of 0.78. The reliability of the research tool (questionnaire) was also tested by a Cronbach’s alpha analysis to create indexes for the questionnaire. We created the following indexes: Locus of control (7 items; α = 0.69), Self-efficacy (19 items; α = 0.71), Concerns (4 items; α = -0.79), Exposure to information (3 items; α = -0.82), Subjective norms (2 items; α = -0.76), Reported actual earthquake preparation (7 items, α = -0.76).

### Analysis

A research team of three researchers analyzed the qualitative research using the thematic analysis method [45]. Relevant themes and sub-themes were extracted concerning perceptions regarding earthquakes. To ensure reliability, two members of the research team carried out independent analyses of the data. Each began the analysis by reading the transcripts to extract general and potential meanings. Then, each created an initial coding structure based on descriptive coding resulting from coding units of text as themes by labeling them with a phrase related to the participant’s account. In the next stage, we conducted a joint analysis and consolidated the identified themes.

In the experimental research, we used the following steps to analyze the data: First, we tested the success of the experimental manipulation, namely the validity of dividing the participants into high-risk and low-risk residential areas and examining their absorption of new information. The manipulation’s success was tested by comparing the responses of participants who received new information to those of participants who did not receive new information. Participants answered the following open question before and after receiving the information: “To the best of your knowledge, what are the factors that determine the magnitude of an earthquake in a particular place and the damage it will cause?” To analyze the data, we classified the participants’ answers into the following categories: building strength, location, distance from the sea, soil type, earthquake magnitude, and earthquake preparedness. Before participants were given the new information, there were no significant differences between the two groups.

Before being given any information or guidelines about earthquake preparation, the interviewees were also asked: "Have you already done anything or are you planning to do anything to protect yourself and your family from the impact and damage of a major earthquake that may hit Israel?” Possible answers were: “I have done something”; “I have not done anything but plan to do something soon”; “I haven’t done anything but plan to do something at some point”; and “I haven’t done anything and do not intend to.”

Second, we built an index of willingness to prepare for an earthquake. This index included a general question about willingness to prepare for an earthquake as well as questions about specific behavioral steps, such as stockpiling water and/or food, conducting regular earthquake drills with household members, removing objects hanging on walls and searching different sources for earthquake forecast information. Cronbach’s alpha of this index was 0.76, indicating that all the specific items are intercorrelated and can be combined into one common index. The subsequent analysis was carried out on this common index.

Third, we compared the variables associated with behavioral willingness to prepare for an earthquake before the experiment manipulation with the variables contributing to preparedness after the exposure. We used an F test to identify the variables contributing to earthquake preparedness that emerged from the stage prior to the experimental manipulation. In the third stage we used an F test to identify the variables associated with behavioral willingness after the experimental manipulation.

### Ethics

The study was approved by the Committee on Health and Welfare Sciences, The Faculty of Social Welfare and Health Sciences at the University of Haifa, approval number 304/18. All the study participants gave their written consent to participate in the research and publish its results.

## Results

### Qualitative findings

In the interviews, the experts estimated that a major earthquake will occur in Israel, though it is impossible to know when, where or at what magnitude. The formula used to calculate earthquake magnitude is a matter of dispute, leading most experts to discuss a reference scenario rather than a forecast scenario. Despite their reluctance, we asked experts to indicate what earthquake magnitude Israel should be preparing for. Most indicated a magnitude of 7.5 on the Richter scale, though some claimed that Israel should also prepare for an 8-magnitude earthquake. Furthermore, contrary to the public’s perception of high-risk and low-risk areas for earthquakes, some experts stated that the factors determining earthquake intensity and damage are building strength, earthquake magnitude, distance from earthquake epicenter, and soil type. Table 2 outlines the interview findings and provides selected quotes from the experts.

**Table 2:** Interviews with the experts: Findings and selected quotes regarding the variables associated with earthquake occurrence (n = 19).

Finding	Agreement/Lack of Agreement	Selected Quotations
**Chances of earthquake occurrence in Israel and earthquake magnitude**	All interviewees indicate an earthquake will definitely occur in Israel, but they cannot predict when, where or at what magnitude	“*The bottom line is that there is going to be an earthquake in Israel*, *but within that there is a lot of uncertainty*.*”*
**Reference scenario rather than forecast scenario**	Dispute between a reference scenario of 7.5 magnitude and 8.0 magnitude.	“*Some experts say we should prepare for an 8-magnitude earthquake*, *whereas the prevailing opinion is 7*.*5-magnitude*. *We must prepare for an 8-magnitude earthquake*, *which means all buildings must be reinforced*.*”*
	Full consensus that a major earthquake will constitute a national-scale disaster with catastrophic consequences.	
		“*Ultimately we can’t know whether an event of this sort will happen in my lifetime or that of my great grandchildren*. *It can happen any minute*… *Therefore*, *we cannot live according to the worst scenario*. *The real question is the frequency of serious events*. *Preparation ultimately means allocating the appropriate resources*. *So it is actually an arbitrary decision by one person*… *who must attempt to reasonably defend the decision*. *Just like in the case of an army that must be prepared for any attack*, *and in the case of road safety*, *it is also the case for earthquakes*.*”*
**Factors influencing earthquake occurrence**	Experts list four variables that determine earthquake magnitude and damage	*“Earthquake frequency*, *magnitude*, *size and expected damage are matters of dispute”*
		“*The factors influencing earthquake magnitude and damage differ from the public’s perceptions*. *People think that geographic location is what matters and believes there are high-risk and low-risk areas*. *This perception is inaccurate*. *Additional factors come into play*: *building strength*, *earthquake magnitude*, *distance from earthquake epicenter*, *soil type*.”

### Quantitative findings

The sociodemographic characteristics of the participants are presented in S1 Table.

#### The experimental manipulation

The validity of the division into high-risk and low-risk areas was tested using the following statement: “There is a chance that a significant earthquake will occur in my area of residence.” Respondents answered on a scale ranging from 1 to 7, where 7 indicated a high degree of agreement with the statement (Table 3).

**Table 3:** Respondents’ assessments of chances an earthquake will occur in their area of residence (n = 834).

Statement	Respondents’ assessment (1 = Low degree of agreement; 7 = High degree of agreement	Area’s earthquake occurrence risk category		
		High-risk	Low-risk	Total
		n (%)	n (%)	n (%)
There is a chance a significant earthquake will occur in my area of residence	1	12 (3.3)	51 (10.9)	63 (7.6)
	2	16 (4.3)	57 (12.2)	73 (8.8)
	3	27 (7.3)	62 (13.3)	89 (10.7)
	4	66 (17.9)	77 (16.5)	143 (17.1)
	5	52 (14.1)	53 (11.4)	105 (12.6)
	6	62 (16.8)	28 (6.0)	90 (10.8)
	7	76 (20.7)	23 (4.9)	99 (11.9)
	Don’t know	57 (15.5)	115 (24.7)	172 (20.6)
Average (*P* < 0.001, *χ*^2^_(1)_ = 113.1)		5.0	3.6	

Among the respondents with a high level of agreement (5–7 range), 51.6% lived in areas categorized as high-risk, compared to 22.3% who lived in areas categorized as low-risk. The chi square test result was significant (*P* < 0.001, *χ*^2^_(1)_ = 102.1).

After the participants were given the new information, the information group exhibited significantly higher percentages on each category than the group that did not receive the information. One exception was the advance preparation category, where no differences emerged between the groups. For all the other categories, the percentage of responses in the group that received information was significantly higher than the corresponding percentage in the group that did not receive information (Table 4).

**Table 4:** Factors that determine the magnitude of an earthquake in a particular place and the damage it will cause (n = 834).

Factor	Experimental group		*P*	*χ*^2^_(1)_
	Received new information (%)	Did not receive information (%)		
Construction quality	53.4	36.3	<0.001	25.2
Proximity to the sea	52.3	24.0	<0.001	24.0
Soil type	28.0	0.7	<0.001	122.0
Earthquake magnitude	86.6	59.2	<0.001	78.6

#### Variables correlated with preparedness

*Before the experimental manipulation*. Correlation with area of residence: An analysis of the correlations among a number of the sociodemographic characteristics, including area of residence, age, gender, income level and nationality, indicated an effect of area of residence, such that the average willingness to prepare (on a 4-point scale) was 2.017 in low-risk areas compared to 2.232 in high-risk areas (F(1,832) = 5.756, p = .017). Moreover, an interaction was found between area of residence and nationality, such that Arabs living in low-risk areas were more willing to prepare than Jews living in these areas: 2.358 versus 1,961 (F(1,464) = 5.459, p = .020).

*Impact of subjective norms on correlation with areas of residence*. The following question was used to examine the perceived level of earthquake preparation norms: “How many of the people in your social environment do you think have prepared or intend to prepare for a major earthquake?” Even when the variables of subjective norm and socio-cognitive characteristics (e.g., external or internal locus of control, efficacy) are added to the sociodemographic characteristics, the norm outweighs them all. Namely, the only statistically significant effect of each variable on its own is that of the norm. For example, the average willingness to prepare rises from 1.85 among those indicating that very few of their friends have prepared or intend to prepare to 2.188 among those reporting this for a small number of their friends. This figure further rises to 2.442 among those who report that a moderate number of their friends have prepared or will prepare and to 2.885 among those who report this for a fairly large number of their friends (F(3,757) = 42.796, p = .000). In addition, the interaction between norm and area has an impact, such that the discrepancy between high-risk and low-risk areas remains only when the norm is very low (i.e., participants perceived the norm only applied to a very small segment of their social environment). In this case, the average willingness to prepare was 1.755 in low-risk areas compared to 1.936 in high-risk areas (F(1,422) = 4.507, p = 0.343).

*After the experimental manipulation*. After the experimental manipulation, participants were asked the same question about willingness to prepare, with the following introductory statement: “Before we end, we would like to repeat a few of the questions…” The findings show that after the experimental manipulation, the effect of geographical area (high or low-risk) disappeared, while an effect of the interaction between area and new information emerged (Table 5). Among people living in low-risk areas, those who were given new information exhibited greater willingness to prepare than those who did not receive the new information. Among those living in high-risk areas, the new information had the opposite effect: the level of willingness to prepare among those given the new information was lower than among those who were not given the information. No effect emerged for the communicator.

**Table 5:** Average (on a 4-point scale) willingness to prepare for an earthquake after the experimental manipulation, according to residential area(n = 834).

Experimental manipulation	Area’s earthquake occurrence risk category	
	High-risk	Low-risk
	average (n)	average (n)
Received new information	2.20 (190)	2.67 (231)
Did not received new information	2.40 (178)	2.22 (235)
Total	2.30 (368)	2.44 (466)

F(2,664) = 3.297, p = .038.

## Discussion

The research findings indicate that among all the socioeconomic and residential attributes examined, the variable with the greatest influence on willingness to prepare for an earthquake is geographical area of residence. Israel is located along the Great Rift Valley and for that reason has been hit by a number of earthquakes in the past that caused grave damage to life and property. Recently, Israel has been hit by several low magnitude earthquakes. Earthquake experts believe it is almost inevitable that Israel will be hit by a powerful earthquake that could claim thousands of lives and cause serious damage to property and buildings. Some estimate that a major earthquake occurs in this area every 70 to 100 years [33, 46, 47]. Indeed, it is commonly acknowledged that a high magnitude earthquake will hit areas of Israel located along the Rift (hereinafter: high-risk areas).

Contrary to common knowledge according to which the primary factor determining the intensity of any earthquake in Israel is geographical area, the experts interviewed for this study pointed to additional factors that influence earthquake intensity and damage: building strength, earthquake magnitude, distance from the earthquake epicenter, soil type, and the interaction between these four factors. The research findings indicate that being informed of this expert information (referred to in the experiment as new information) affects the public’s willingness to prepare for future earthquakes. The direction of this influence depends on area of residence and extent to which the new information is consistent with common knowledge. In areas that are considered at low-risk according to common knowledge, the new and added information increased people’s willingness to prepare. Conversely, in areas that are at high-risk according to common knowledge, the new and added information actually decreased willingness.

According to the Sandman model [21], risk perception, both in the general public and among experts, consists of information about the hazard and the emotional element, namely concern and outrage. Based on this model, the discrepancy between expert estimation of earthquake risk and that of the public can be explained as follows. When the perceived level of concern over earthquakes is low among people living in areas considered low-risk while at the same time the level of concern among experts is high) as supported by the interviews with the experts), a gap emerges between the public and the experts. According to Sandman, in order to reduce this gap the public must be given relevant information to raise its level of concern. The experimental findings indicate that the gap between the views of those living in so-called low-risk areas and those of the experts was mitigated when we gave the public new information. That is, by pointing out additional variables that can affect earthquake magnitude and the resultant damage, we likely raised participants’ level of concern. Indeed, we can assume that participants who thought they were “safe” based on common knowledge changed their attitudes and became more willing to prepare.

One might expect that the new information would impact willingness to prepare for earthquakes among those living in high-risk areas as well. Yet contrary to expectations, among those living in areas considered high-risk according to common knowledge, the added information reduced their willingness to prepare. One possible explanation for this finding is as follows: Over the years people living in high-risk areas believed they were at higher risk of earthquakes than others. The new information showed them their risk was no higher than that of people living in the allegedly low-risk areas. This discovery apparently reduced their sense of concern and consequently affected their willingness to prepare. This finding exposes the risk of a boomerang effect [23, 24] in these areas. After receiving new information people are less willing to prepare, contrary to the declared goal of the authorities to encourage preparedness. How then can the authorities deal with or prevent such a boomerang effect?

## Conclusions

The results of this study point to the important role played by norms. As the experimental findings show, subjective perceptions of norms affect people’s willingness to prepare for earthquakes more than any other variable. Hence, in order for expert information to become common knowledge and perceived by the public as the norm, the authorities must launch an information campaign and a widespread and ongoing public intervention program to provide the public with the new information. The goal is to create a new norm that will affect the preparation willingness and actual preparation of all the residents of Israel, regardless of where they live.

To counter the boomerang effect, experts and the media must engage in more effective and transparent communication with the public. The authorities must implement the inoculation theory [25], according to which an attitude or a belief can be safeguarded against persuasion or influence in much the same way a body can be protected against disease, for example through pre-exposure to weakened versions of a more serious future threat [25, 26].

## Limitations and future research

This study has some limitations. The experiment was conducted under laboratory conditions, with high internal validity but low external validity. This research requires ongoing examination, including the development of explanatory variables for willingness to prepare and actual preparedness for earthquakes. Follow-up studies can involve field experiments in localities commonly considered to be high-risk areas compared to those thought to be low-risk areas to test the actual impact of “new information" over time.

## Supporting information

S1 Appendix The experiment questionnaire English version. (PDF)

S2 Appendix The experiment questionnaire Hebrew version. (PDF)

S1 Table Sociodemographic characteristics and earthquake risk in residential area (n = 834). (DOCX)
