# Supplementary material for: The influence of new information that contradicts common knowledge about earthquake preparedness in Israel: A mixed methods experiment study
Source: PLoS One. 2021 Apr 14;16(4):e0250127. doi: 10.1371/journal.pone.0250127 (PMC8046234; doi:10.1371/journal.pone.0250127)
Supplement: S1 Appendix — (PDF) [file pone.0250127.s001.pdf]

S1 Appendix: The experiment questionnaire English version

Q1: What is your sex?

1 __ Male
2 __ Female

Q2: What is your age?

1 __ 17 or under
2 __ 18-24
3 __ 25-34
4 __ 35-44
5 __ 45-54
6 __ 55-64
7 __ 65+

Entrance FOR Q3: If Q1[any] Skip to Q4

Q3: Are you:

1 __ Muslim
2 __ Christian
3 __ Druze
4 __ Other

Q4: Residential Area:

1 __ Metulla area: Hula Valley, Kiryat Shmona, Tel Hai, Sde Eliezer, Kfar Baruch
2 __ Gilboa area: Avital, Adirim, Beit Hashita, Gan Ner, Beit Alfa, Dvora, Teibe
3 __ Nazareth: Kfar Hahoresh, Mizra, Yifat, Genigar, Ramat David, Beit Rimon, Alonim
4 __ Safed area: Elifelet, Shefer, Kfar Shamai, Rosh Pina, Safsufa, Meron
5 __ Tiberias area: Kinneret, Degania, Haon, Beit Zera, Ashdot Yaakov, Amiad, Ginosar, Kfar Hitim
6 __ Jordan Valley and Beit Shean: Bikot, Argeman, Jericho, Patsael, Shdemot Mehula, Shluchot, Reshafim, Sde Trumot, Tirat Zvi
7 __ Haifa Bay area: All neighborhoods in Haifa—Hadar, Carmel, Ahuza, Denia—plus Kiryat Haim, Kiryat Yam
8 __ Dead Sea area: Naaran, Beit Haarava, Almog, Avnit, Mitspeh Shalem, Rotem
9 __ Arava area: Neot Hakikar, Ein Yahav, Sapir, Tsofar, Faran
10 __ Eilat area: Eilot, Elifaz, Yotvata, Grofit, Lotan
11 __ Tel Aviv and Dan area
12 __ Northern Sharon area
13 __ Other locality in the Galilee not mentioned above
14 __ Judea and Samaria
15 __ Jerusalem and surrounding area
16 __ Negev and southern area
17 __ Other

Q5: Allocation

1 __ Hit by earthquakes
2 __ Not hit by earthquakes

Q6: You are being asked to participate in an important research study on the topic of public preparation for earthquakes. Your answers will help in determining policy for preparing the public in the event of an earthquake. Please note that this is an experiment that replicates actual situations in preparing for earthquakes in Israel. In your opinion, what is the probability that a strong earthquake will hit Israel within the next five years?

1 __ 100% certain that this will occur
2 __ Extremely high probability
3 __ High probability
4 __ Somewhat high probability
5 __ Moderate probability
6 __ Somewhat low probability
7 __ Low probability
8 __ Extremely low probability
9 __ An earthquake will not occur (zero probability)
10 __ Don’t know

Q7: Are you concerned or not concerned that a strong earthquake will hit Israel within the next five years?

1 __ Concerned
2 __ A bit concerned
3 __ Not very concerned
4 __ Not at all concerned
5 __ Don’t know

Q8: From time to time experts declare that a strong earthquake is likely to occur in Israel within the next five years. Do you know the probability that a strong earthquake will hit Israel within the next five years according to most of the experts?

1 __ 100% certain that this will occur
2 __ Extremely high probability
3 __ High probability
4 __ Somewhat high probability
5 __ Moderate probability
6 __ Somewhat low probability
7 __ Low probability
8 __ Extremely low probability
9 __ An earthquake will not occur (zero probability)
10 __ Don’t know

Q9: If a strong earthquake hits Israel, what do you believe is the probability that you or someone in your family will be hurt?

1 __ Extremely high probability
2 __ High probability
3 __ Somewhat high probability
4 __ Moderate probability
5 __ Somewhat low probability
6 __ Low probability
7 __ Extremely low probability
8 __ Don’t know

Q10: If a strong earthquake hits Israel, what do you believe is the probability of major damage to your property?

1 __ Extremely high probability
2 __ High probability
3 __ Somewhat high probability
4 __ Moderate probability
5 __ Somewhat low probability
6 __ Low probability
7 __ Extremely low probability
8 __ Don’t know

Q11: Based on what you know, what are the factors that determine the intensity with which an earthquake hits a particular location and the damage caused?

Q12: To what extent do you agree or disagree with each of the following statements:

	1 Totally Agree	2 Somewhat Agree	3 Somewhat Disagree	4 Totally Disagree	5 Don’t Know
12-1: I trust the experts from the field of earthquakes and believe that their forecasts about the probability that a strong earthquake will hit Israel are correct	__	__	__	__	__
12-2: I think that even the experts cannot know whether or not a strong earthquake will hit Israel in the near future	__	__	__	__	__

Q13: Based on your impression, are the experts providing the public all the information the public needs regarding earthquakes or not?

1 __ Providing all the information
2 __ Providing most of the information /a significant amount of information
3 __ Providing only a small portion of the information
4 __ Not providing any information at all
5 __ Don't know

Q14: Have you been exposed to information that explains how to prepare in advance for a strong earthquake?

1 __ I have been exposed to sufficient information
2 __ I have been exposed to some information, although not enough
3 __ I have not been exposed to any information on this topic
4 __ Don't know

Q15: Have you been exposed to information that explains how to behave during a strong earthquake?

1 __ I have been exposed to sufficient information
2 __ I have been exposed to some information, although not enough
3 __ I have not been exposed to any information on this topic
4 __ Don't know

Entrance FOR Q16: If Q14[(A1)|(A2)] | Q15 [(A1)|(A2)] Skip to Q16 ELSE Skip to Q17

Q16: Indicate all the sources that provided you with information about earthquakes:

1 __ Website of the Homefront Command (RND)
2 __ Social networks (RND)
3 __ Ministry of Education (RND)
4 __ Television (RND)
5 __ Radio (RND)
6 __ Print journalism (RND)
7 __ Internet (RND)
8 __ Other people (RND)
9 __ Other. Provide details.

10 __ Don't remember (MUL)

Q17: Israel must allocate major resources to its security. The national budget is limited and adding money to one area comes at the expense of other areas. In your opinion, what priority should be given to preparing for a strong earthquake?

1 __ Very high
2 __ High
3 __ Somewhat high
4 __ Somewhat low
5 __ Low
6 __ Very low

7 __ Don’t know

Q18: In your opinion, what organization should have the main administrative responsibility in case a strong earthquake hits Israel? Indicate what in your opinion should be the main organization.

1 __ Local government authority in each locality (RND)
2 __ Homefront Command (RND)
3 __ National Emergency Management Authority (NEMA) (RND)
4 __ Police (RND)
5 __ Fire Department (RND)
6 __ Other. Provide details.

7 __ Don’t know

Q19: Who should be the main spokesperson on matters related to preparing the public for a strong earthquake?

1 __ Homefront Command Spokesperson (RND)
2 __ Spokesperson of local authority in each locality (RND)
3 __ Prime Minister’s Office Spokesperson (RND)
4 __ National Emergency Management Authority (NEMA) Spokesperson (RND)
5 __ Expert from the Geological Institute (RND)
6 __ Don’t know

Q20: Based on your impression, who is actually the spokesperson?

1 __ Homefront Command Spokesperson (RND)
2 __ Spokesperson of local authority in each locality (RND)
3 __ Prime Minister’s Office Spokesperson (RND)
4 __ National Emergency Management Authority (NEMA) Spokesperson (RND)
5 __ Expert from the Geological Institute (RND)
6 __ Citizens on social networks (RND)
7 __ No one
8 __ Don’t know

Q21: In your opinion, who should assume the main responsibility for reinforcing residential structures to withstand a strong earthquake?

1 __ Homeowners (RND)
2 __ Local government authority (RND)
3 __ National government (RND)
4 __ National Emergency Management Authority (RND)
5 __ Amidar (RND)
6 __ Other. Provide details.

7 __ Don’t know

Q22: Based on your impression, does the media give the topic of preparing the public for a major earthquake the appropriate amount of attention, too much attention or not enough attention.

1 __ Much too much attention
2 __ Too much attention
3 __ An appropriate amount of attention
4 __ Too little attention
5 __ Much too little attention
6 __ Don’t know

Q23: Based on your impression, to what extent is the education system preparing students with respect to earthquakes?

1 __ Much too much preparation
2 __ Too much preparation
3 __ An appropriate amount of preparation
4 __ Too little preparation
5 __ Much too little preparation
6 __ Don’t know

Q24: Based on your impression, has the local government authority in your place of residence prepared the residents with respect to proper behavior in the case of an earthquake?

1 __ Much too much preparation
2 __ Too much preparation
3 __ An appropriate amount of preparation
4 __ Too little preparation
5 __ Much too little preparation
6 __ Don’t know

Q25: Based on your impression, how is the Homefront Command dealing with the matter of preparing the public for a major earthquake: appropriately, too much or not enough?

1 __ Much more than necessary
2 __ More than necessary
3 __ Appropriately
4 __ Less than necessary
5 __ Much less than necessary
6 __ Don’t know

Q26: Are the current instructions given to citizens regarding how to behave during an earthquake clear enough or not clear enough?

1 __ I have not received any instructions
2 __ The instructions are not at all clear
3 __ Some of the instructions are clear and some are not
4 __ Most or all of the instructions are not clear
5 __ Don’t know

Q27: Until now have you done anything or are you planning to do anything to protect yourself and your family from being harmed by a strong earthquake, if one should hit Israel?

1 __ Already done something
2 __ Have not done anything but plan to do so in the near future
3 __ Have not done anything but plan to do so in the distant future
4 __ Have not done anything and don't plan to do anything
5 __ Don't know

Entrance FOR Q28: IF Q27[(A1)] Skip to Q28 ELSE Skip to Q29

Q28: Please indicate all the steps you have taken to protect yourself and your family.

Q29: For each of the following actions, please indicate if you have already done it, plan to do it, or have not done it and do not plan to do it:

	1 Already done it	2 Haven't done it but plan to do it soon	3 Haven't done it but plan to do it eventually	4 Haven't done it and don't plan to do it	5 Don't Know
29-1: Stockpile food and/or water	__	__	__	__	__
29-2: Reinforce the building foundations and/or the supporting pillars	__	__	__	__	__
29-3: Practice steps to be taken during an earthquake on a regular basis with everyone in the family, including small children	__	__	__	__	__
29-4: Purchase emergency lighting	__	__	__	__	__
29-5: Remove items hanging over beds or reinforce items hanging on the walls	__	__	__	__	__
29-6: Search websites in Israel and worldwide for information about how to behave during an earthquake	__	__	__	__	__
29-7: Prepare a first-aid kit	__	__	__	__	__
29-8: Take out homeowners insurance for earthquake damage	__	__	__	__	__

Q30: Has the building where you live been reinforced to withstand earthquakes?

1 __ Reinforced
2 __ Not reinforced
3 __ I don't know

Entrance FOR Q31: IF Q30[(A2)|(A3)] Skip to Q31 ELSE Skip to Q32

Q31: Do you intend to take steps to ensure that the building in which you live is reinforced to withstand earthquakes?

1 __ Plan to take steps in the near future
2 __ Plan to take steps in the distant future
3 __ Do not plan to take steps
4 __ Don’t know

Q32: Has the school/kindergarten building where your children are enrolled been reinforced to withstand earthquakes?

1 __ Reinforced
2 __ Not reinforced
3 __ I don’t know
4 __ I do not have any children in school

Entrance FOR Q33: IF Q32[(A2)|(A3)] Skip to Q33 ELSE Skip to Q34

Q33: Do you intend to take steps to insure that the school/kindergarten building where your children are enrolled is reinforced to withstand earthquakes?

1 __ Plan to take steps in the near future
2 __ Plan to take steps in the distant future
3 __ Do not plan to take steps
4 __ Don’t know

Q34: Based on your impression, what proportion of people in your social environment have prepared or intend to prepare for a strong earthquake?

1 __ A very large proportion
2 __ A large proportion
3 __ A somewhat large proportion
4 __ A somewhat small proportion
5 __ A small proportion
6 __ A very small proportion
7 __ Don’t know

Q35: Based on your impression, what proportion of people in the general public have prepared or intend to prepare for a strong earthquake?

1 __ A very large proportion
2 __ A large proportion
3 __ A somewhat large proportion
4 __ A somewhat small proportion
5 __ A small proportion
6 __ A very small proportion
7 __ Don’t know

Q36: Do you know enough about what you should do during the first moments of a major earthquake?

1 __ I know enough
2 __ I know something but not enough
3 __ I don't know anything

Q37: For each of the following statements, please indicate whether you believe it is correct or incorrect:

	1<br>I'm sure it's correct	2<br>I think it's correct	3<br>I think it's not correct	4<br>I'm sure it's not correct	5<br>Don't Know
37-1: We cannot afford the expense of reinforcing our building to withstand a major earthquake	—	—	—	—	—
37-2: Some of the neighbors in my building do not agree to spend the money necessary to reinforce it.	—	—	—	—	—
37-3: The city has not approved the Tama 38 Reinforcement Plan for the building where I live	—	—	—	—	—
37-4: Removing pictures from the wall as recommended in preparing for a major earthquake will harm the appearance of my apartment	—	—	—	—	—
37-5: We have no room to store stockpiled food and/or water	—	—	—	—	—
37-6: I don't want to think about earthquakes. The thought of an earthquake only makes me feel bad.	—	—	—	—	—
37-7: If people discover we are stockpiling food to prepare for an earthquake, they will make fun of us.	—	—	—	—	—
37-8: As part of earthquake preparation, it's important to check the mezuzahs in the house and make sure they are kosher	—	—	—	—	—

Q38: To what extent do you agree or disagree with each of the following statements? Please answer on a scale of 1-7, where 1 indicates that you totally disagree and 7 indicates that you totally agree, with the other numbers representing intermediate rankings:

38-1: Every adult citizen must see himself or herself as having sole responsibility for preparing family members for a major earthquake:

1 2 3 4 5 6 7

__ Don’t know

38-2: Minor actions such as removing pictures from the walls and stockpiling food and water will have major consequences for what happens to family members in the case of a major earthquake:

1 2 3 4 5 6 7

__ Don’t know

38-3: The main thing that will affect family members in the case of a major earthquake in our area of residence is whether we already have taken steps to prepare:

1 2 3 4 5 6 7

__ Don’t know

38-4: Whatever we do will not make any difference. What determines what happens to the family in the case of an earthquake is only the intensity of the earthquake and the local conditions:

1 2 3 4 5 6 7

__ Don’t know

38-5: Whatever we do will not make any difference. In the case of an earthquake, only God/fate will determine what happens to me and my family:

1 2 3 4 5 6 7

__ Don’t know

38-6: Preparing for a major earthquake will be effective in reducing damages:

1 2 3 4 5 6 7

__ Don’t know

38-7: If I prepare in advance for a major earthquake, the chances I will be hurt are lower:

1 2 3 4 5 6 7

__ Don’t know

38-8: Preparations for a major earthquake are easy to carry out:

1 2 3 4 5 6 7

__ Don’t know

38-9: I already have the possibility of preparing for a major earthquake:

1 2 3 4 5 6 7

__ Don’t know

38-10: I believe that a major earthquake poses a serious threat to the public:

1 2 3 4 5 6 7

__ Don’t know

38-11: I believe that a major earthquake will cause serious damages to the public:

1 2 3 4 5 6 7

__ Don’t know

38-12: The area where I live is considered a high-risk area for a major earthquake:

1 2 3 4 5 6 7

__ Don’t know

38-13: The chances are high that a major earthquake will occur in my area of residence:

1 2 3 4 5 6 7

__ Don’t know

Q39: Until this point you have answered questions on the topic of earthquakes. We now want to provide you the information we were given by the Homefront Command spokesperson and then ask you a number of questions related to this information. Please read the message from the Homefront Command spokesperson at least twice before you answer the questions.
“Hello, I am the Homefront Command Spokesperson and I want to talk to you about preparing for an earthquake in Israel. In the past there have been destructive earthquakes in our region, and the occurrence of another one is only a matter of time. It is certain that an earthquake will occur, but we don’t know when or where. For years many geologists in Israel and worldwide have been attempting to develop tools to predict earthquakes. These geologists have also attempted to calculate the chances of an earthquake occurring in a particular region and at what intensity. Yet as I’ve already mentioned, despite these major efforts over the years by the world’s top geologists, it is still impossible to predict when, where and at what intensity an earthquake will occur. Accumulated experience across the globe proves that citizens’ advance preparation for earthquakes and their proper conduct during earthquakes have saved many lives and reduced property damage. I want to offer you a number of guidelines for advanced preparation for earthquakes that the Homefront Command has prepared: a) Building strength—The best way to prevent loss of lives and property is to make sure that the building in which you live can withstand an earthquake; b) Preparing the interior of your home, mainly by removing pictures or shelves above beds; c) Reinforcing solar water heaters and other water heaters; d) Storing toxic or flammable substances under lock and key and far away from sources of heat; e) Determining in advance the safest place in your home during an earthquake; f) Preparing emergency equipment for the family (food and water, emergency lighting, first aid kit, medications and the like); g) Practicing closing the main gas tap and electricity switch; h) Informing everyone in the family what to do in the case of an earthquake, and practicing with everyone, including small children, what each person needs to do in case of an earthquake. This practice should take place on a regular basis, so that it becomes a habit, just like putting on a seatbelt whenever you get into the car.

1 __ Continuation

Q40: Was the information provided by the Homefront Command spokesperson clear or not clear?

1 __ Totally clear
2 __ Somewhat clear
3 __ Somewhat unclear
4 __ Totally unclear

Q41: Based on what you know, what are the factors that determine the intensity of an earthquake at a particular location and the damage it will cause?

Q42: In conclusion, we want to repeat a number of questions. Do you intend to take any steps to protect yourself and your family from harm and damages caused by a major earthquake, if one occurs in Israel?

1 __ Plan to take steps in the near future
2 __ Plan to take steps in the distant future
3 __ Do not plan to take steps
4 __ Don't know

Q43: For each of the following actions, please indicate if you plan to do it or do not plan to do it. If you have already taken this action, please indicate this:

	1 Plan to do it soon	2 Plan to do it eventually	3 Haven't done it and don't plan to do it	4 Already did it	5 Don't Know
43-1: Stockpile food and/or water	__	__	__	__	__
43-2: Reinforce building foundations and/or supporting pillars	__	__	__	__	__
43-3: Practice steps to be taken during an earthquake on a regular basis with everyone in the family, including small children	__	__	__	__	__
43-4: Purchase emergency lighting	__	__	__	__	__
43-5: Remove items hanging over beds or reinforce items hanging on the walls	__	__	__	__	__
43-6: Search websites in Israel and worldwide for information about how to behave during an earthquake	__	__	__	__	__
43-7: Prepare a first-aid kit	__	__	__	__	__
43-8: Take out homeowners insurance for earthquake damage	__	__	__	__	__

Entrance FOR Q44: IF Q30[(A2)|(A3)] Skip to Q44 ELSE Skip to Q45

Q44: Do you intend to take steps to ensure that the building in which you live is reinforced to withstand earthquakes?

1 __ Plan to take steps in the near future
2 __ Plan to take steps in the distant future
3 __ Do not plan to take steps
4 __ Don't know

Entrance FOR Q45: IF Q32[(A2)|(A3)] Skip to Q45 ELSE Skip to Q46

Q45: Do you intend to take steps to insure that the school/kindergarten building where your children are enrolled is reinforced to withstand earthquakes?

1 __ Plan to take steps in the near future
2 __ Plan to take steps in the distant future

3 __ Do not plan to take steps
4 __ Don’t know

Q46: Were you personally or was anyone close to you present at any place in Israel during an earthquake that was strong enough to be felt?

1 __ Yes, I was
2 __ Yes. Someone close to me but not me
3 __ No. Not me and not anyone close to me
4 __ Don’t know

Q47: Were you personally or was anyone close to you physically present somewhere at a time when an earthquake occurred or shortly thereafter?

1 __ Yes, I was
2 __ Yes. Someone close to me but not me
3 __ No. Not me and not anyone close to me
4 __ Don’t know

Q48: To what extent do you agree or disagree with each of the following statements? Please answer on a scale of 1-7, where 1 indicates that you totally disagree and 7 indicates that you totally agree, with the other numbers representing intermediate rankings:

48-1: Every adult citizen must see himself or herself as having sole responsibility for preparing family members for a major earthquake:

1 2 3 4 5 6 7

48-2: The chances are high that a major earthquake will occur in my area of residence:

1 2 3 4 5 6 7

Q49: If a strong earthquake hits Israel, what do you believe is the probability that you or someone in your family will be hurt?

1 __ Extremely high probability
2 __ High probability
3 __ Somewhat high probability
4 __ Moderate probability
5 __ Somewhat low probability
6 __ Low probability
7 __ Extremely low probability
8 __ Don’t know

Q50: If a strong earthquake hits Israel, what do you believe is the probability of major damage to your property?

1 __ Extremely high probability
2 __ High probability
3 __ Somewhat high probability
4 __ Moderate probability
5 __ Somewhat low probability
6 __ Low probability

7 __ Extremely low probability
8 __ Don’t know

Q51: Based on your impression, are the experts providing the public all the information the public needs regarding earthquakes or not?
1 __ Providing all the information
2 __ Providing most of the information /a significant amount of information
3 __ Providing only a small portion of the information
4 __ Not providing any information at all
5 __ Don’t know

Q52: In your opinion, what organization should have the main responsibility in case a strong earthquake hits Israel? Indicate what in your opinion should be the main organization.
1 __ Local government authority in each locality (RND)
2 __ Homefront Command (RND)
3 __ National Emergency Management Authority (NEMA) (RND)
4 __ Police (RND)
5 __ Fire Department (RND)
6 __ Other. Provide details.
7 __ Don’t know

Q53: Who should be the main spokesperson on matters related to preparing the public for a strong earthquake?
1 __ Homefront Command Spokesperson (RND)
2 __ Spokesperson of local authority in each locality (RND)
3 __ Prime Minister’s Office Spokesperson (RND)
4 __ National Emergency Management Authority (NEMA) Spokesperson (RND)
5 __ Expert from the Geological Institute (RND)
6 __ Don’t know

Q54: Do you believe it is possible to take preparatory actions to reduce the chances of being injured by a strong earthquake?
1 __ Believe it is possible
2 __ Tend to believe it is possible
3 __ Tend not to believe it is possible
4 __ Do not believe it is possible
5 __ Don’t know

Q55: How old are you?

Q56: How do you define your religious observance?
1 __ Secular
2 __ Traditional
3 __ Religious

4 __ Ultra-Orthodox
5 __ Other

Q57: The average monthly gross family income in Israel is 13,5000 shekels (if you live alone, the average gross monthly income for an individual is 7,950 shekels). Please rank your family income relative to the average:
1 __ Much below average
2 __ Somewhat below average
3 __ Average
4 __ Somewhat above average
5 __ Much above average
6 __ Refuse to answer

Q58: What is your level of education?
1 __ Elementary / some high school
2 __ High school
3 __ Post-secondary
4 __ Academic
5 __ Refuse to answer

Q59: Where were you born?
1 __ Israel
2 __ Former Soviet Union
3 __ Other
4 __ Refuse to answer

Entrance FOR Q60: IF Q59[(A2)] Skip to Q60 ELSE Skip to Q61

Q60: Did you immigrate before or after 1990?
1 __ Before 1990
2 __ 1990 or later

Q61: Where was your father born?
1 __ Israel
2 __ Former Soviet Union
3 __ Other
4 __ Refuse to answer

Q62: Which of the following statements is correct regarding the apartment in which you live:
1 __ The apartment is owned by me or by my parents
2 __ The apartment is owned by a relative
3 __ The apartment is rented / owned by Amidar / key money
4 __ Refuse to answer

Q63: When was the building where you live built?
1 __ Before 1980
2 __ 1980 and after

3 __ Don’t know

Q64: What is the name of the locality where you live?

Q65: Has the building where you live been reinforced to withstand earthquakes?

1 __ Reinforced
2 __ Not reinforced
3 __ Don’t know

Q66: Has the school/kindergarten where your children are enrolled been reinforced to withstand earthquakes?

1 __ Reinforced
2 __ Not reinforced
3 __ Don’t know
4 __ I do not have children in school

Q67: Based on what you know, what are the factors that determine the intensity of an earthquake at a particular location and the damage it will cause?

Q68: Have you done anything or are you planning to do anything to protect yourself and your family from harm and damages caused by a strong earthquake, if one should hit Israel?

1 __ Already done something
2 __ Have not done anything but plan to do so in the near future
3 __ Have not done anything but plan to do so in the distant future
4 __ Have not done anything and don’t plan to do anything
5 __ Don’t know

Q69: For each of the following actions, please indicate if you have already done it, plan to do it, or have not done it and do not plan to do it:

	1 Already done it	2 Haven’t done it but plan to do it soon	3 Haven’t done it but plan to do it eventually	4 Haven’t done it and don’t plan to do it	5 Don’t Know
69-1: Stockpile food and/or water	__	__	__	__	__
69-2: Reinforce the building foundations and/or the supporting pillars	__	__	__	__	__
69-3: Practice steps to be taken during an earthquake on a regular basis with everyone in the family, including small children	__	__	__	__	__

69-4: Purchase emergency lighting	__	__	__	__	__
69-5: Remove items hanging over beds or reinforce items hanging on the walls	__	__	__	__	__
69-6: Search websites in Israel and worldwide for information about how to behave during an earthquake	__	__	__	__	__
69-7: Prepare a first-aid kit	__	__	__	__	__
69-8: Take out homeowners insurance for earthquake damage	__	__	__	__	__

Q70: Do you intend to take steps to ensure that the building in which you live is reinforced to withstand earthquakes?

1 __ Plan to take steps in the near future
2 __ Plan to take steps in the distant future
3 __ Do not plan to take steps
4 __ Don’t know

Q71: Do you intend to take steps to insure that the school/kindergarten building where your children are enrolled is reinforced to withstand earthquakes?

1 __ Plan to take steps in the near future
2 __ Plan to take steps in the distant future
3 __ Do not plan to take steps
4 __ Don’t know

Q72: Were you personally or was anyone close to you present at any place in Israel during an earthquake that was strong enough to be felt?

1 __ Yes, I was
2 __ Yes. Someone close to me but not me
3 __ No. Not me and not anyone close to me
4 __ Don’t know

Q73: Were you personally or was anyone close to you physically present somewhere at a time when an earthquake occurred or shortly thereafter?

1 __ Yes, I was
2 __ Yes. Someone close to me but not me
3 __ No. Not me and not anyone close to me
4 __ Don’t know

Q74: To what extent do you agree or disagree with each of the following statements? Please answer on a scale of 1-7, where 1 indicates that you totally disagree and 7 indicates that you totally agree, with the other numbers representing intermediate rankings:

74-1: Every adult citizen must see himself or herself as having sole responsibility for preparing family members for a major earthquake:

1 2 3 4 5 6 7

__ Don’t know

74-2: The chances are high that a major earthquake will occur in my area of residence:

1 2 3 4 5 6 7

__ Don’t know

Q75: If a strong earthquake hits Israel, what do you believe is the probability that you or someone in your family will be hurt?

1 __ Extremely high probability
2 __ High probability
3 __ Somewhat high probability
4 __ Moderate probability
5 __ Somewhat low probability
6 __ Low probability
7 __ Extremely low probability
8 __ Don’t know

Q76: If a strong earthquake hits Israel, what do you believe is the probability of major damage to your property?

1 __ Extremely high probability
2 __ High probability
3 __ Somewhat high probability
4 __ Moderate probability
5 __ Somewhat low probability
6 __ Low probability
7 __ Extremely low probability
8 __ Don’t know

Q77: Based on your impression, are the experts providing the public all the information the public needs regarding earthquakes or not?

1 __ Providing all the information
2 __ Providing most of the information /a significant amount of information
3 __ Providing only a small portion of the information
4 __ Not providing any information at all
5 __ Don’t know

Q78: In your opinion, what organization should have the main responsibility in case a strong earthquake hits Israel? Indicate what in your opinion should be the main organization.

1 __ Local government authority in each locality (RND)
2 __ Homefront Command (RND)
3 __ National Emergency Management Authority (NEMA) (RND)
4 __ Police (RND)
5 __ Fire Department (RND)
6 __ Other. Provide details.

7 __ Don’t know

Q79: Who should be the main spokesperson on matters related to preparing the public for a strong earthquake?

1 __ Homefront Command Spokesperson (RND)
2 __ Spokesperson of local authority in each locality (RND)
3 __ Prime Minister’s Office Spokesperson (RND)
4 __ National Emergency Management Authority (NEMA) Spokesperson (RND)
5 __ Expert from the Geological Institute (RND)
6 __ Don’t know

Q80: Do you believe it is possible to take preparatory actions to reduce the chances of being injured by a strong earthquake?

1 __ Believe it is possible
2 __ Tend to believe it is possible
3 __ Tend not to believe it is possible
4 __ Do not believe it is possible
5 __ Don’t know
