# Supplementary material for: The influence of new information that contradicts common knowledge about earthquake preparedness in Israel: A mixed methods experiment study
Source: PLoS One. 2021 Apr 14;16(4):e0250127. doi: 10.1371/journal.pone.0250127 (PMC8046234; doi:10.1371/journal.pone.0250127)
Supplement: S2 Appendix — (PDF) [file pone.0250127.s002.pdf]

S2 Appendix: The experiment questionnaire Hebrew version

Q 1 מהו מינך?

1 ○ גבר
2 ○ אישה

Q 2 מהו גילך?

1 ○ 17 ומטה
2 ○ 18-24
3 ○ 25-34
4 ○ 35-44
5 ○ 45-54
6 ○ 55-64
7 ○ +65

Entrance FOR Q3: IF Q1[any] Skip to Q4

Q 3 האם אתה:

1 ○ מוסלמי
2 ○ נוצרי
3 ○ דרוזי
4 ○ אחר

Q 4 אזור מגורים:

1 ○ **<u>יישובים באזור מטולה:</u>** עמק החולה, קרית שמונה, תל חי, שדה אליעזר, כפר ברוך
2 ○ **<u>יישובים באזור הגלבוע:</u>** אביטל, אדירים, בית השיטה, גן נר, בית אלפא, דבורה, טייבה
3 ○ **<u>נצרת:</u>** כפר החורש, מזרע, יפעת, גניגר, רמת דוד, בית רימון, אלונים
4 ○ **<u>יישובים באזור צפת:</u>** אליפלט, שפר, כפר שמאי, ראש פינה, ספסופה, מירון
5 ○ **<u>יישובים ליד טבריה:</u>** כנרת, דגניה, האון, בית זרע, אשדות יעקוב, עמיעד, גנוסר, כפר חיטים
6 ○ **<u>הבקעה ובית שאן:</u>** בקעות, ארגמן, יריחו, פצאל, שדמות מחולה, שלוחות, רשפים, שדי תרומות, טירת צבי
7 ○ **<u>מפרץ חיפה:</u>** כלל השכונות בחיפה- הדר, כרמל, אחוזה, דניה..בנוסף קרית חיים, קרית ים
8 ○ **<u>ים המלח:</u>** נערן, בית הערבה, אלמוג, אבנת, מצפה שלם, רותם
9 ○ **<u>ערבה:</u>** נאות הכיכר, עין יהב, ספיר, צופר, פארן
10 ○ **<u>אילת:</u>** אילות, אליפז, יטבתה, גרופית, לוטן
11 ○ אזור תל אביב וגוש דן
12 ○ השרון הצפוני
13 ○ ישוב אחר בגליל שלא מוזכר
14 ○ יהודה ושומרון
15 ○ ירושליים והסביבה
16 ○ אזור הנגב והדרום
17 ○ אחר

Entrance FOR Q5: IF Q4[(A1)|(A2)|(A3)|(A4)|(A5)|(A6)|(A7)|(A8)|(A9)|(A10)] Set (A=1) ELSEIF Q4[(A11)|(A12)|(A13)|(A14)|(A15)|(A16)|(A17)] Set (A=2)

Q 5 מכסה

1. מוכה רעידות אדמה
2. לא מוכה רעידות אדמה

Q 6

שלום,אתם משתתפים במחקר חשוב בנושא של היערכות הציבור לרעידת אדמה. התשובות שלכם תעזורנה בקביעת תוכנית להכנת הצבור למקרה של רעידת אדמה. חשוב לציין שמדובר בניסוי בלבד שמדמה מצבים אמיתיים להערכות לרעידת אדמה בישראל. מה לדעתך הסיכוי שבחמש השנים הקרובות תתרחש רעידת אדמה חזקה בישראל?

1. וודאות של מאה אחוז שתתרחש
2. סיכוי גבוה מאוד
3. סיכוי גבוה
4. סיכוי די גבוה
5. סיכוי בינוני
6. סיכוי די נמוך
7. סיכוי נמוך
8. סיכוי נמוך מאוד
9. לא תתרחש רעידת אדמה (סיכוי אפסי)
10. לא יודע

Q 7

האם אתה חושש, או לא חושש, שבחמש השנים הבאות תתרחש בישראל רעידת אדמה חזקה?

1. חושש
2. חושש מעט
3. די לא חושש
4. כלל לא חושש
5. לא יודע

Q 8

המומחים מודיעים מדי פעם שיש סיכויים שבישראל תתרחש רעידת אדמה חזקה בשנים הקרובות. האם אתה יודע, מהם הסיכויים לרעידת אדמה חזקה לפי ההערכות של רוב המומחים?

1. וודאות של מאה אחוז שתתרחש
2. סיכוי גבוה מאד
3. סיכוי גבוה
4. סיכוי די גבוה
5. סיכוי בינוני
6. סיכוי די נמוך
7. סיכוי נמוך
8. סיכוי נמוך מאוד
9. לא תתרחש רעידת אדמה (סיכוי אפסי)
10. לא יודע

Q 9

אם תתרחש רעידת אדמה חזקה בישראל, מה לדעתך הסיכוי שאתה או מישהו מבני ביתך תפגעו, חלילה?

1. סיכוי גבוה מאוד
2. סיכוי גבוה
3. סיכוי די גבוה
4. סיכוי בינוני
5. סיכוי די נמוך
6. סיכוי נמוך
7. סיכוי נמוך מאוד
8. לא יודע

Q 10

אם תתרחש רעידת אדמה חזקה בישראל, מה, לדעתך, הסיכוי שתהיה חלילה פגיעה משמעותית ברכוש שלך?

1. סיכוי גבוה מאוד

2 ◯ סיכוי גבוה
3 ◯ סיכוי די גבוה
4 ◯ סיכוי בינוני
5 ◯ סיכוי די נמוך
6 ◯ סיכוי נמוך
7 ◯ סיכוי נמוך מאוד
8 ◯ לא יודע

Q 11

ממה שאתה יודע מהם הגורמים שקובעים את עוצמת הפגיעה מרעידת אדמה במקום מסוים ואת הנזקים שהיא תגרום?

Q

באיזו מידה אתה מסכים או לא מסכים עם כל אחד מהמשפטים הבאים:

	1 מסכים בהחלט	2 די מסכים	3 די לא מסכים	4 כלל לא מסכים	5 לא יודע
12-1 אני סומך על המומחים בתחום רעידות אדמה שהתחזיות שלהם לגבי הסיכוי שתתרחש רעידת אדמה חזקה בישראל נכונות	◯	◯	◯	◯	◯
12-2 אני חושב שגם המומחים לא יכולים לדעת אם תתרחש או לא תתרחש בעתיד הקרוב רעידת אדמה חזקה בישראל	◯	◯	◯	◯	◯

Q 13

ממה שאתה מתרשם, האם המומחים מעבירים או לא מעבירים לציבור את כל המידע שהציבור צריך לדעת לגבי רעידות אדמה?

1 ◯ מעבירים את כל המידע
2 ◯ מעבירים את רוב המידע/ חלק משמעותי מהמידע
3 ◯ מעבירים רק חלק קטן מהמידע
4 ◯ בכלל לא מעבירים מידע
5 ◯ לא יודע

Q 14

האם נחשפת למידע המסביר כיצד להיערך מראש לרעידת אדמה חזקה?

1 ◯ נחשפתי למידע מספיק
2 ◯ נחשפתי למידע , אך לא מספיק
3 ◯ כלל לא נחשפתי למידע בעניין
4 ◯ לא יודע

Q 15

האם נחשפת למידע כיצד לנהוג בעת התרחשות רעידת אדמה חזקה?

1 ◯ נחשפתי למידע מספיק
2 ◯ נחשפתי למידע , אך לא מספיק
3 ◯ כלל לא נחשפתי למידע בעניין
4 ◯ לא יודע

Entrance FOR Q16: IF Q14[(A1)|(A2)] | Q15[(A1)|(A2)] Skip to Q16 ELSE Skip to Q17

Q 16

ציין את כל המקורות דרכם נחשפת למידע בנושא רעידת אדמה:

1 ☐ אתר פיקוד העורף (RND)
2 ☐ רשתות חברתיות (RND)
3 ☐ משרד החינוך (RND)
4 ☐ טלוויזיה (RND)
5 ☐ רדיו (RND)
6 ☐ עיתונות כתובה (RND)
7 ☐ אינטרנט (RND)
8 ☐ מאנשים אחרים (RND)
9 ☐ אחר. פרט

10 ☐ לא זוכר (MUL)

Q 17

ישראל היא מדינה שחייבת להקצות משאבים רבים לנושא הביטחון; תקציב המדינה מוגבל ותוספת תקציב לסעיף מסוים בא על חשבון הסעיפים האחרים. באיזה סדר עדיפות צריך, לדעתך, להיות התקציב של הערכות לרעידת אדמה חזקה?

1 ○ גבוה מאד
2 ○ גבוה
3 ○ די גבוה
4 ○ די נמוך
5 ○ נמוך
6 ○ נמוך מאד
7 ○ לא יודע

Q 18 (1 [illegible]) (52,78)

איזה גוף, לדעתך, צריך להיות האחראי העיקרי על ניהול ההערכות לרעידת אדמה חזקה, אם תתרחש בישראל? ציין גוף אחד עיקרי לדעתך

1 ○ הרשויות המקומיות, בכל ישוב הרשות המקומית של הישוב (RND)
2 ○ פיקוד העורף (RND)
3 ○ רשות חרום לאומית (רח"ל) (RND)
4 ○ משטרה (RND)
5 ○ מכבי אש (RND)
6 ○ אחר -פרט

7 ○ לא יודע

(53,79) (1 [illegible])

Q 19 מי צריך להיות הדובר העיקרי לציבור בנושאים הקשורים להערכות הציבור לרעידת אדמה חזקה?

1 ○ דובר פיקוד העורף (RND)
2 ○ בכל ישוב דוברות הרשות המקומית (RND)
3 ○ דובר משרד ראש הממשלה (RND)
4 ○ דובר רשות החרום הלאומית(רח"ל) (RND)
5 ○ מומחה מטעם המכון הגיאולוגי (RND)
6 ○ לא יודע

Q 20 וממה שאתה מתרשם, מיהו הדובר בפועל?

1 ○ דובר פיקוד העורף (RND)
2 ○ בכל ישוב דוברות הרשות המקומית (RND)
3 ○ דובר משרד ראש הממשלה (RND)
4 ○ דובר רשות החרום הלאומית(רח"ל) (RND)
5 ○ מומחה מטעם המכון הגיאולוגי (RND)
6 ○ אזרחים ברשתות החברתיות (RND)
7 ○ אף אחד
8 ○ לא יודע

Q 21

מי, לדעתך, צריך להיות האחראי העיקרי על חיזוק מבני המגורים של האזרחים כדי שיעמדו ברעידת אדמה חזקה אם תתרחש?

1 ○ בעלי הדירות/בתים (RND)
2 ○ הרשות המקומית (RND)
3 ○ הממשלה (RND)
4 ○ רשות חרום לאומית (RND)
5 ○ עמידר (RND)
6 ○ אחר- פרט

7 ○ לא יודע

Q 22

ממה שאתה מתרשם, האם התקשורת עוסקת בנושא של הערכות הציבור לרעידת אדמה גדולה במידה המתאימה, יותר מידי או פחות מידי?

1 ○ עוסקת הרבה יותר מידי
2 ○ עוסקת יותר מידי
3 ○ עוסקת במידה המתאימה
4 ○ עוסקת פחות מידי
5 ○ עוסקת הרבה פחות מידי
6 ○ לא יודע

Q 23

ממה שאתה מתרשם, האם מערכת החינוך מכינה את התלמידים להתנהגות מתאימה בכל הקשור להערכות לקראת רעידת אדמה ?

1 ○ מכינה הרבה יותר מידי
2 ○ מכינה יותר מידי
3 ○ מכינה במידה המתאימה
4 ○ מכינה פחות מידי
5 ○ מכינה הרבה פחות מידי
6 ○ לא יודע

Q 24

ממה שאתה מתרשם, האם הרשות המקומית בישוב מגוריך מכינה את התושבים להתנהגות מתאימה בכל הקשור להערכות לקראת רעידת אדמה?

1 ○ מכינה הרבה יותר מידי
2 ○ מכינה יותר מידי
3 ○ מכינה במידה המתאימה
4 ○ מכינה פחות מידי
5 ○ מכינה הרבה פחות מידי
6 ○ לא יודע

Q 25

ממה שאתה מתרשם, האם פיקוד העורף מטפל בנושא של הערכות הצבור לרעידת אדמה גדולה במידה המתאימה, יותר מידי, או פחות מידי?

1 ○ מטפל הרבה יותר מהדרוש
2 ○ מטפל יותר מידי
3 ○ מטפל במידה המתאימה
4 ○ מטפל פחות מידי
5 ○ מטפל הרבה פחות מהדרוש
6 ○ לא יודע

Q 26

האם ההנחיות להתנהגות בעת רעידת אדמה, כפי שהן ניתנות היום לאזרחים, מספיק ברורות או לא מספיק ברורות?

1 ◯ לא נחשפתי להנחיות
2 ◯ ההנחיות כלל לא ברורות
3 ◯ חלק מההנחיות ברורות וחלק לא ברורות
4 ◯ רוב או כל ההנחיות לא ברורות
5 ◯ לא יודע

Q 27 (1 ספ') (42,68)

האם עד כה עשית משהו או מתכנן לעשות משהו כדי להגן עליך ועל בני ביתך מפני נזקים ופגיעות של רעידת אדמה חזקה , אם תתרחש בישראל?

1 ◯ עשיתי | 4
2 ◯ עדיין לא עשיתי, אך מתכנן לעשות בטווח הקרוב | 3
3 ◯ לא עשיתי, אך מתכנן לעשות בטווח הרחוק | 2
4 ◯ לא עשיתי ולא מתכנן לעשות | 1
5 ◯ לא יודע | אין קוד

Entrance FOR Q28: IF Q27[(A1)] Skip to Q28 ELSE Skip to Q29

Q28 ציין, בבקשה, את כל הפעולות שעשית כדי להגן עליך ועל בני ביתך:

Q 29 (1 ספ') (43,69)

לגבי כל אחד מהדברים הבאים ציין בבקשה האם עשית אותו, או שאתה מתכנן לעשות אותו, או שלא עשית אותו ואינך מתכנן לעשות אותו:
(שורה)

		1 עשיתי	2 לא עשיתי אך מתכנן לעשות בזמן הקרוב	3 לא עשיתי אך מתכנן לעשות בטווח הרחוק	4 לא עשיתי ולא מתכנן לעשות	5 לא יודע
		4	3	2	1	ס/ו קוד
29_1	הצטיידות במזון ו/או מים	◯	◯	◯	◯	◯
29_2	חיזוק יסודות הבית ו/או ריסון קומת העמודים	◯	◯	◯	◯	◯
29_3	תרגול, דרך קבע כל כמה זמן, עם בני הבית, גם עם הילדים הקטנים, תוך כדי ביצוע פעולות אותן כל בן בית יצטרך לבצע במקרה של רעידת אדמה	◯	◯	◯	◯	◯
29_4	קניית תאורת חירום	◯	◯	◯	◯	◯
29_5	הורדת חפצים שתלויים מעל המיטה או חיזוק חפצים שתלויים על הקירות	◯	◯	◯	◯	◯
29_6	חיפוש מידע באתרים בארץ ובעולם לגבי ההתנהגות בעת רעידת אדמה	◯	◯	◯	◯	◯
29_7	הכנת ערכת עזרה ראשונה	◯	◯	◯	◯	◯
29_8	ביטוח יחידת המגורים למקרה של פגיעה כתוצאה מרעידת אדמה	◯	◯	◯	◯	◯

Q 30

האם המבנה בו אתה גר מחוזק נגד רעידת אדמה?

1 ◯ מחוזק
2 ◯ לא מחוזק
3 ◯ לא ידוע לי

Entrance FOR Q31: IF Q30[(A2)|(A3)] Skip to Q31 ELSE Skip to Q32

Q 31

האם אתה מתכוון לנקוט בצעדים שיבטיחו שהמבנה בו אתה גר יהיה מחוזק נגד רעידת אדמה?

1 ◯ מתכוון לנקוט צעדים בעתיד הקרוב
2 ◯ מתכוון לנקוט צעדים בטווח הרחוק
3 ◯ לא מתכוון לנקוט צעדים
4 ◯ לא יודע

Q 32

האם בית הספר/גן ילדים בו לומדים ילדיך מחוזק נגד רעידת אדמה?

1 ◯ מחוזק
2 ◯ לא מחוזק
3 ◯ לא ידוע לי
4 ◯ אין לי ילדים במערכת החינוך

Entrance FOR Q33: IF Q32[(A2)|(A3)] Skip to Q33 ELSE Skip to Q34

Q 33

האם אתה מתכוון או לא מתכוון, לנקוט בצעדים שידאגו לכך שבית הספר/ גן הילדים בו לומדים ילדיך, יהיה מחוזק נגד רעידת אדמה?

1 ◯ מתכוון לנקוט צעדים בזמן הקרוב
2 ◯ מתכוון לנקוט צעדים בטווח הרחוק
3 ◯ לא מתכוון לנקוט צעדים
4 ◯ לא יודע

Q 34

ממה שאתה מתרשם, איזה חלק מהאנשים בסביבתך החברתית עשה או מתכוון לעשות הכנות לקראת רעידת אדמה חזקה?

1 ◯ חלק גדול מאוד
2 ◯ חלק גדול
3 ◯ חלק די גדול
4 ◯ חלק די קטן
5 ◯ חלק קטן
6 ◯ חלק קטן מאוד
7 ◯ לא יודע

Q 35

ממה שאתה מתרשם, איזה חלק מהאנשים בציבור הרחב עשה או מתכוון לעשות הכנות לקראת רעידת אדמה חזקה?

1 ◯ חלק גדול מאוד
2 ◯ חלק גדול
3 ◯ חלק די גדול
4 ◯ חלק די קטן
5 ◯ חלק קטן
6 ◯ חלק קטן מאוד
7 ◯ לא יודע

Q 36

האם אתה יודע מספיק או לא יודע מספיק, מה עליך לעשות ברגעים הראשונים של רעידת אדמה חזקה?

1 יודע מספיק
2 יודע אבל לא מספיק
3 כלל לא יודע

Q לגבי כל אחת מהאמירות הבאות ציין, בבקשה, אם היא נכונה או לא נכונה, לדעתך.

	1 בטוח שנכונה	2 חושב שנכונה	3 חושב שלא נכונה	4 בטוח שלא נכונה	5 לא יודע
37-1 איננו יכולים לעמוד בהוצאה הכרוכה בחיזוק בתים כדי שהבניין שלנו יעמוד ברעידת אדמה משמעותית	○	○	○	○	○
37.2 בבניין שלי יש שכנים שלא מסכימים להוציא את הכסף הנדרש לחיזוק המבנה	○	○	○	○	○
37-3 העיריה לא מאשרת את יישום תכנית תמ"א 38 לבניין שאני גר בו	○	○	○	○	○
37-4 הורדת תמונות מהקיר כפי שמומלץ להכנות לקראת רעידת אדמה משמעותית תפגע בחזות הדירה	○ 4	○ 3	○ 2	○ 1	○ 0
37-5 אין לנו מקום אחסון שנדרש לצורך אגירת מזון ו/מים	○	○	○	○	○
37-6 אני לא רוצה לחשוב על רעידת אדמה; המחשבה על רעידת אדמה עושה לי לא טוב	○ 4	○ 3	○ 2	○ 1	○ 0
37-7 אם נאגור מזון למקרה של רעידת אדמה ואנשים ידעו על כך הם ילעגו לנו	○ 4	○ 3	○ 2	○ 1	○ 0
37-8 חשוב לבדוק את המזוזות בבית ולוודא שהן כשרות כחלק מהערכות לרעידת אדמה	○	○	○	○	○

Q עד כמה אתה מסכים, או לא מסכים, עם כל אחת מהאמירות הבאות?תן את תשובתך בסולם בן 7 דרגות, כאשר: "1" - מציין שאתה כלל לא מסכים עם האמירה ו-"7" - מציין שאתה מסכים עם האמירה במידה רבה מאוד. השאר הן דרגות ביניים:

כל אזרח בוגר צריך לראות את עצמו כאחראי הבלעדי להיערכות של בני ביתו למקרה שתתרחש רעידת אדמה גדולה

1 2 3 4 5 6 7

לא יודע ☐

(1 [illegible]) 38-1 (48-1, 74-1)

לפעולות קטנות, כמו הסרת תמונות מהקירות, הכנת מזון ומים תהיינה השלכות משמעותיות על מצבם של בני ביתי אם תתרחש רעידת אדמה משמעותית

1 2 3 4 5 6 7

לא יודע ☐

38-2

הדבר העיקרי שישפיע על מצבם של בני ביתי אם תתרחש רעידת אדמה משמעותית באזור מגורינו הם צעדי ההערכות אם ננקוט אותם כבר עכשיו

1 2 3 4 5 6 7

38-3

לא יודע

לא משנה מה אעשה, מה שיקבע את מצבם של בני ביתי זה רק עוצמת רעידת האדמה אם תקרה ותנאי המקום 38-4

1 2 3 4 5 6 7

לא יודע

לא משנה מה אעשה, אלוהים/הגורל יקבעו את המצב שלי/ של בני ביתי, אם תתרחש רעידת אדמה 38-5

1 2 3 4 5 6 7

לא יודע

הערכות לקראת רעידת אדמה משמעותית אם תקרה יעילה בהפחתת הנזקים 38-6

1 2 3 4 5 6 7

לא יודע

אם איערך לקראת רעידת אדמה חזקה יקטן הסיכוי שאפגע אם היא תתרחש 38-7

1 2 3 4 5 6 7

לא יודע

פעולות למען הערכות לרעידת אדמה חזקה אם תתרחש הן פעולות קלות לביצוע 38-8

1 2 3 4 5 6 7

לא יודע 0

7 6 5 4 3 2 1

יש באפשרותי להיערך כבר עכשיו למקרה שתתרחש רעידת אדמה חזקה 38-9

1 2 3 4 5 6 7

לא יודע

אני מאמין שרעידת אדמה משמעותית היא איום חמור לציבור 38-10

1 2 3 4 5 6 7

לא יודע

אני מאמין שרעידת אדמה משמעותית תגרום נזקים חמורים לצבור 38-11

1 2 3 4 5 6 7

לא יודע

האזור בו אני גר נחשב כאזור בסיכון גבוה לרעידת אדמה משמעותית 38-12

1 2 3 4 5 6 7

לא יודע

יש סיכוי גבוה שבאזור המגורים שלי תתרחש רעידת אדמה משמעותית. (1 fr) 38-13

1 2 3 4 5 6 7

לא יודע

(48-2, 74-2)

Q 39

עד כה ענית על שאלות שנוגעות לנושא של רעידת אדמה. אנחנו רוצים להעביר לך כעת את הדברים שהעביר אלינו דובר פיקוד העורף ואחר כך להציג לך מספר שאלות בקשר לכך. אנא קרא לפחות פעמיים את דברי דובר פיקוד העורף ואחרי זה השב לשאלות. "שלום, אני דובר פיקוד העורף ואני רוצה לדבר אתכם על היערכות לקראת התרחשות של רעידת אדמה בישראל.בעבר אירעו באזורנו רעידות אדמה הרסניות והתרחשות רעידה נוספת היא רק שאלה של זמן. יש ודאות שרעידת אדמה תתרחש אבל לא ידוע מתי ואיפה. במשך השנים גאולוגים רבים בישראל ובעולם כולו מנסים לפתח כלים לחיזוי רעידת אדמה. הגיאולוגים מנסים גם לנסות לחשב לאזורים שונים את הסיכויים שתתרחש רעידת אדמה באזור ובאיזו עוצמה. אבל, כפי שכבר שאמרנו, למרות העבודה המאומצת במשך שנים רבות של מיטב הגאולוגים בעולם עדיין לא ניתן לחזות מתי רעידת אדמה תתרחש באיזה מקום ובאיזו עוצמה. הניסיון שנצבר בעולם מוכיח שהיערכות מוקדמת של התושבים לקראת רעידת אדמה והתנהלות נכונה בזמן התרחשותה, הצילו חיים רבים ומזערו נזק לרכוש. אני רוצה להציג לך, כמה הנחיות להערכות מוקדמת לרעידת אדמה שהוציא פיקוד העורף: א. עמידות הבניין. הדרך המשמעותית ביותר למנוע אבדות בנפש וברכוש היא לדאוג שהמבנה בו גרים יעמוד בפני רעידות אדמה.ב. הכנת פנים הבית; בעיקר הסרת תמונות או מדפים מעל המיטות.ג. תמיכה בדודי שמש ודודי חימום אחרים.ד. אחסון חומרים רעילים או דליקים במקום נעול הרחק ממקור חימום.ה. קביעה מראש של מקום בטוח, עד כמה שאפשר, בבית.ו. הכנת ציוד חרום משפחתי (מזון ומים, תאורת חרום, ערכת עזרה ראשונה, תרופות וכו')ז. תרגול סגירת ברז הגז ומפסק החשמל הראשיים.ח. העברת מידע לבני הבית מה לעשות במקרה שתתרחש רעידת אדמהט. תרגול עם בני הבית, גם עם הילדים הקטנים, תוך כדי ביצוע פעולות אותן כל בן בית יצטרך לבצע במקרה של רעידת אדמה. התרגול צריך להתבצע דרך קבע כל כמה זמן, כך שייעשה להרגל כמו למשל- חגירת חגורת בטיחות בכניסה למכונית.

1 ◯ המשך

Q 40

האם המידע שהעביר דובר פיקוד העורף, היה מובן או לא מובן?

1 ◯ מובן לגמרי
2 ◯ די מובן
3 ◯ די לא מובן
4 ◯ כלל לא מובן

Q 41

ממה שאתה יודע מהם הגורמים שקובעים את עוצמת הפגיעה מרעידת אדמה במקום מסוים ואת הנזקים שהיא תגרום?

Q 42

לקראת סיום, אנחנו רוצים להציג לך מספר שאלות חוזרות האם אתה מתכוון או לא מתכוון לעשות משהו בכדי להגן עליך ועל בני ביתך מפני נזקים ופגיעות של רעידת אדמה חזקה, אם תתרחש בישראל?

1 ◯ מתכוון לעשות בטווח הקרוב
2 ◯ מתכוון לעשות בטווח הרחוק
3 ◯ לא מתכוון לעשות
4 ◯ לא יודע

Q

לגבי כל אחד מהדברים הבאים ציין בבקשה אם מתכוון לעשות אותו, או שאינך מתכוון לעשות אותו. אם כבר ביצעת את הדבר, ציין זאת.

	1 מתכוון לעשות בזמן הקרוב	2 מתכוון לעשות בטווח הרחוק	3 לא עשיתי ולא מתכוון לעשות	4 כבר ביצעתי זאת	5 לא יודע
43-1 הצטיידות במזון ו/או מים	◯	◯	◯	◯	◯

	1 מתכוון לעשות בזמן הקרוב	2 מתכוון לעשות בטווח הרחוק	3 לא עשיתי ולא מתכוון לעשות	4 כבר ביצעתי זאת	5 לא יודע
43-2 חיזוק יסודות הבית ו/או ריסון קומת העמודים	○	○	○	○	○
43-3 תרגול, דרך קבע כל כמה זמן, עם בני הבית, גם עם הילדים הקטנים, תוך כדי ביצוע פעולות אותן כל בן בית יצטרך לבצע במקרה של רעידת אדמה	○	○	○	○	○
43-4 קניית תאורת חירום	○	○	○	○	○
43-5 הורדת חפצים שתלויים מעל המיטה או חיזוק חפצים שתלויים על הקירות	○	○	○	○	○
43-6 חיפוש מידע באתרים בארץ ובעולם לגבי ההתנהגות בעת רעידת אדמה	○	○	○	○	○
43-7 הכנת ערכת עזרה ראשונה	○	○	○	○	○
43-8 ביטוח יחידת המגורים למקרה של פגיעה כתוצאה מרעידת אדמה	○	○	○	○	○

Entrance FOR Q44: IF Q30[(A2)|(A3)] Skip to Q44 ELSE Skip to Q45

Q 44 (31,70) (2 ד׳)

האם אתה מתכוון או לא מתכוון לנקוט בצעדים שידאגו לכך שהמבנה בו אתה גר יהיה מחוזק נגד רעידת אדמה

1 ○ מתכוון לנקוט בעתיד הקרוב
2 ○ מתכוון לנקוט בטווח הרחוק
3 ○ לא מתכוון לנקוט הצעדים
4 ○ לא יודע

Entrance FOR Q45: IF Q32[(A2)|(A3)] Skip to Q45 ELSE Skip to Q46

Q 45 (33,71) (2 ד׳)

האם אתה מתכוון או לא מתכוון לנקוט בצעדים שידאגו לכך שהמבנה בו לומדים ילדיך יהיה מחוזק נגד רעידת אדמה?

1 ○ מתכוון לנקוט בטווח הקרוב
2 ○ מתכוון לנקוט בטווח הרחוק
3 ○ לא מתכוון לנקוט
4 ○ לא יודע

Q 46 (72) (1 ד׳)

האם אתה אישית, או מישהו קרוב אליך, הייתם במקום כלשהו בישראל בעת שקרתה בו רעידת אדמה בעוצמה שהורגשה בסביבה?

1 ○ כן, אני
2 ○ כן, מישהו קרוב אלי אך לא אני עצמי
3 ○ לא אני ולא מישהו קרוב אלי
4 ○ לא יודע

Q 47

(73) (1 גר)

האם אתה, אישית, או מישהו קרוב אליך נוכחתם פיזית, במקום שקרתה בו רעידת אדמה בעת או בזמן קצר לאחר התרחשותה ?

1 ○ כן, אני
2 ○ כן, מישהו קרוב אלי, אך לא אני עצמי
3 ○ לא אני ולא מישהו קרוב אלי
4 ○ לא יודע

Q

עד כמה אתה מסכים, או לא מסכים, עם כל אחת מהאמירות הבאות?(תן את תשובתך בסולם בין 7 דרגות, כאלה "1"- מציין שאתה כלל לא מסכים עם האמירה ו-"7"- מציין כי אתה מסכים אם האמירה במידה רבה מאוד. השאר הן דרגות ביניים). (38,74)

כל אזרח בוגר, צריך לראות את עצמו כאחראי הבלעדי להערכות של בני ביתו למקרה שתתרחש רעידת אדמה גדולה. (2 גר) 48-1

1	2	3	4	5	6	7	
							☐ לא יודע

יש סיכוי גבוה שבאזור המגורים שלי תתרחש רעידת אדמה משמעותית. (2 גר) 48-2

1	2	3	4	5	6	7	
							☐ לא יודע

Q 49 (2 גר) (9,75)

אם תתרחש רעידת אדמה חזקה בישראל, מה לדעתך הסיכוי שאתה או מישהו מבני ביתך תפגעו, חלילה?

1 ○ סיכוי גבוה מאוד 7
2 ○ סיכוי גבוה 6
3 ○ סיכוי די גבוה 5
4 ○ סיכוי בינוני 4
5 ○ סיכוי די נמוך 3
6 ○ סיכוי נמוך 2
7 ○ סיכוי נמוך מאוד 1
8 ○ לא יודע 0

Q 50 (2 גר) (10,76)

אם תתרחש רעידת אדמה חזקה בישראל, מה, לדעתך, הסיכוי שתהיה חלילה פגיעה משמעותית ברכוש שלך?

1 ○ סיכוי גבוה מאוד
2 ○ סיכוי גבוה
3 ○ סיכוי די גבוה
4 ○ סיכוי בינוני
5 ○ סיכוי די נמוך
6 ○ סיכוי נמוך
7 ○ סיכוי נמוך מאוד
8 ○ לא יודע

Q 51 (2 גר) (13,77)

ממה שאתה מתרשם, האם המומחים מעבירים או לא מעבירים לציבור את כל המידע שהציבור צריך לדעת לגבי רעידות אדמה?

1 ○ מעבירים את כל המידע 4
2 ○ מעבירים את רוב המידע/ חלק משמעותי מהמידע 3
3 ○ מעבירים רק חלק קטן מהמידע 2
4 ○ לא מעבירים כל מידע 1
5 ○ לא יודע 0

(18,78) (2 ל"ר) Q 52

איזה גוף, לדעתך, צריך להיות האחראי העיקרי על ניהול ההערכות לרעידת אדמה חזקה, אם תתרחש בישראל? ציין גוף אחד עיקרי לדעתך

1 הרשויות המקומיות, בכל ישוב הרשות המקומית של הישוב (RND)
2 פיקוד העורף (RND)
3 רשות חרום לאומית (רח"ל) (RND)
4 משטרה (RND)
5 מכבי אש (RND)
6 אחר -פרט

7 לא יודע

(19,79) (2 ל"ר)

Q 53 מי צריך להיות הדובר העיקרי לציבור בנושאים הקשורים להערכות הציבור לרעידת אדמה חזקה?

1 דובר פיקוד העורף
2 בכל ישוב דוברות הרשות המקומית
3 דובר משרד ראש הממשלה
4 דובר רשות החרום הלאומית(רח"ל)
5 מומחה מטעם המכון הגיאולוגי
6 לא יודע

---

(80) (1 ל"ר) Q

54 האם אתה מאמין או לא מאמין שניתן לנקוט בפעולות הכנה שיפחיתו את הסיכוי להיפגע מרעידת אדמה חזקה?

1 מאמין
2 נוטה להאמין
3 נוטה לא להאמין
4 כלל לא מאמין
5 לא יודע

Q 55 מהו גילך?

Q 56 איך את/ה מגדיר/ה את עצמך מבחינה דתית?

1 חילוני
2 מסורתי
3 דתי
4 חרדי
5 אחר

Q 57

ההכנסה הממוצעת למשפחה בישראל היא 13,500 ש"ח ברוטו(אם אתה גר לבד, ליחיד 7,950 ש"ח ברוטו)
האם ההכנסה של משפחתך ביחס לממוצע היא:

1 הרבה מתחת לממוצע
2 מעט מתחת לממוצע
3 כמו הממוצע
4 מעט מעל הממוצע
5 הרבה מעל הממוצע
6 מסרב

Q 58 מהי השכלתך?

1 יסודית / תיכונית חלקית
2 תיכונית
3 על תיכונית
4 אקדמאית
5 מסרב

Q היכן נולדת? 59

1 ישראל
2 ברה"מ לשעבר
3 אחר
4 מסרב

Entrance FOR Q60: IF Q59[(A2)] Skip to Q60 ELSE Skip to Q61

Q האם עלית לפני 1990 או אחרי? 60

1 לפני 1990
2 1990 ואילך

Q היכן נולד אביך? 61

1 ישראל
2 ברה"מ לשעבר
3 אחר
4 מסרב

Q מה מהדברים הבאים נכון לגבי הדירה בה אתה גר: 62

1 הדירה בבעלותי או בבעלות הורי
2 הדירה בבעלות קרוב משפחה
3 הדירה שכורה/עמידר/דמי מפתח
4 מסרב

Q מתי נבנה הבניין בו אתה גר- 63

1 לפני 1980
2 1980 ואילך
3 לא יודע

Q מה שם הישוב בו אתה גר? 64

Back

המשך השאלון

Qהאם המבנה בו אתה גר מחוזק נגד רעידת אדמה?

- 1מחוזק
- 2לא מחוזק
- 3לא ידוע לי

Qהאם בית הספר/גן ילדים בו לומדים ילדיך מחוזק נגד רעידת אדמה?

- 1מחוזק
- 2לא מחוזק
- 3לא ידוע לי
- 4אין לי ילדים במערכת החינוך

Qממה שאתה יודע מהם הגורמים שקובעים את עוצמת הפגיעה מרעידת אדמה במקום מסוים ואת הנזקים שהיא תגרום?

Qהאם עד כה עשית משהו או מתכנן לעשות משהו כדי להגן עליך ועל בני ביתך מפני נזקים ופגיעות של רעידת אדמה חזקה , אם תתרחש בישראל?

- 1עשיתי
- 2עדיין לא עשיתי, אך מתכוון לעשות בטווח הקרוב
- 3לא עשיתי, אך מתכוון לעשות בטווח הרחוק
- 4לא עשיתי ולא מתכוון לעשות
- 5לא יודע

לגבי כל אחד מהדברים הבאים ציין בבקשה האם עשית אותו, או שאתה מתכוון לעשות אותו, או שלא עשית אותו ואינך מתכוון לעשות אותו:

(3 ...)

(29, 43)

	1 עשיתי	2 לא עשיתי אך מתכוון לעשות בזמן הקרוב	3 לא עשיתי אך מתכוון לעשות בטווח הרחוק	4 לא עשיתי ולא מתכוון לעשות	5 לא יודע	
הצטיידות במזון ו/או מים	○	○	○	○	○	69-1
חיזוק יסודות הבית ו/או ריסון קומת העמודים	○	○	○	○	○	69-2
תרגול, דרך קבע כל כמה זמן, עם בני הבית, גם עם הילדים הקטנים, תוך כדי ביצוע פעולות אותן כל בן בית יצטרך לבצע במקרה של רעידת אדמה	○	○	○	○	○	69-3
קניית תאורת חירום	○	○	○	○	○	69-4
הורדת חפצים שתלויים מעל המיטה או חיזוק חפצים שתלויים על הקירות	○	○	○	○	○	69-5
חיפוש מידע באתרים בארץ ובעולם לגבי ההתנהגות בעת רעידת אדמה	○	○	○	○	○	69-6
הכנת ערכת עזרה ראשונה	○	○	○	○	○	69-7
ביטוח יחידת המגורים למקרה של פגיעה כתוצאה מרעידת אדמה	○	○	○	○		69-8
	4	3	2	1	[illegible]	

(31,44) (3 ‫ﬠ‬)

Q האם אתה מתכוון לנקוט בצעדים שיבטיחו שהמבנה בו אתה גר יהיה מחוזק נגד רעידת אדמה? 70

1מתכוון לנקוט צעדים בעתיד הקרוב

2מתכוון לנקוט צעדים בטווח הרחוק

3לא מתכוון לנקוט צעדים

4לא יודע

(33,45) (3 ‫ﬠ‬)

Q האם אתה מתכוון או לא מתכוון, לנקוט בצעדים שידאגו לכך שבית הספר/ גן הילדים בו לומדים ילדיך, יהיה מחוזק נגד רעידת אדמה? 71

1מתכוון לנקוט צעדים בזמן הקרוב

2מתכוון לנקוט צעדים בטווח הרחוק

3לא מתכוון לנקוט צעדים

4לא יודע

(46) (2 ‫ﬠ‬)

Q האם אתה אישית, או מישהו קרוב אליך, הייתם במקום כלשהו בישראל בעת שקרתה בו רעידת אדמה בעוצמה שהורגשה בסביבה? 72

1כן, אני

2כן, מישהו קרוב אלי אך לא אני עצמי

3לא אני ולא מישהו קרוב אלי

4לא יודע

(47) (2 ‫ﬠ‬)

Q האם אתה, אישית, או מישהו קרוב אליך נוכחתם פיזית, במקום שקרתה בו רעידת אדמה בעת או בזמן קצר לאחר התרחשותה? 73

1כן, אני

2כן, מישהו קרוב אלי, אך לא אני עצמי

3לא אני ולא מישהו קרוב אלי

4לא יודע

Q עד כמה אתה מסכים, או לא מסכים, עם כל אחת מהאמירות הבאות?(תן את תשובתך בסולם בין 7 דרגות, כאלה "1"- מציין שאתה כלל לא מסכים עם האמירה ו-"7"- מציין כי אתה מסכים אם האמירה במידה רבה מאוד. השאר הן דרגות ביניים).

(3 [illegible])
(38, 48)

כל אזרח בוגר, צריך לראות את עצמו כאחראי הבלעדי להערכות של בני ביתו למקרה שתתרחש רעידת אדמה גדולה.

74-1

1
2
3
4
5
6
7

☐ לא יודע

יש סיכוי גבוה שבאזור המגורים שלי תתרחש רעידת אדמה משמעותית.

74-2

1
2
3
4
5
6
7

☐ לא יודע

Q אם תתרחש רעידת אדמה חזקה בישראל, מה לדעתך הסיכוי שאתה או מישהו מבני ביתך תפגעו, חלילה?

- 1סיכוי גבוה מאוד
- 2סיכוי גבוה
- 3סיכוי די גבוה
- 4סיכוי בינוני
- 5סיכוי די נמוך
- 6סיכוי נמוך
- 7סיכוי נמוך מאוד
- 8לא יודע

Q אם תתרחש רעידת אדמה חזקה בישראל, מה, לדעתך, הסיכוי שתהיה חלילה פגיעה משמעותית ברכוש שלך?

- 1סיכוי גבוה מאוד
- 2סיכוי גבוה
- 3סיכוי די גבוה
- 4סיכוי בינוני
- 5סיכוי די נמוך
- 6סיכוי נמוך
- 7סיכוי נמוך מאוד
- 8לא יודע

Q ממה שאתה מתרשם, האם המומחים מעבירים או לא מעבירים לציבור את כל המידע שהציבור צריך לדעת לגבי רעידות אדמה?

- 1מעבירים את כל המידע
- 2מעבירים את רוב המידע/ חלק משמעותי מהמידע
- 3מעבירים רק חלק קטן מהמידע
- 4לא מעבירים כל מידע
- 5לא יודע

Q איזה גוף, לדעתך, צריך להיות האחראי העיקרי על ניהול ההערכות לרעידת אדמה חזקה, אם תתרחש בישראל? ציין גוף אחד עיקרי לדעתך

1הרשויות המקומיות, בכל ישוב הרשות המקומית של הישוב (RND)

2פיקוד העורף (RND)

3רשות חרום לאומית (רח"ל)(RND))

4משטרה (RND)

5מכבי אש (RND)

6אחר- פרט

7לא יודע

Q מי צריך להיות הדובר העיקרי לציבור בנושאים הקשורים להערכות הציבור לרעידת אדמה חזקה?

79
(3 dk)
(19,53)

1דובר פיקוד העורף

2בכל ישוב דוברות הרשות המקומית

3דובר משרד ראש הממשלה

4דובר רשות החרום הלאומית(רח"ל)

5מומחה מטעם המכון הגיאולוגי

6לא יודע

Q האם אתה מאמין או לא מאמין שניתן לנקוט בפעולות הכנה שיפחיתו את הסיכוי להיפגע מרעידת אדמה חזקה?

80
(2 dk)
(54)

1מאמין

2נוטה להאמין

3נוטה לא להאמין

4כלל לא מאמין

5לא יודע
