# Supplementary material for: The influence of new information that contradicts common knowledge about earthquake preparedness in Israel: A mixed methods experiment study
Source: PLoS One. 2021 Apr 14;16(4):e0250127. doi: 10.1371/journal.pone.0250127 (PMC8046234; doi:10.1371/journal.pone.0250127)
Supplement: S1 Table — (DOCX) [file pone.0250127.s003.docx]

**S1 Table. Sociodemographic characteristics and earthquake risk by residential area (n=834)**

| **Sociodemographic characteristics** | | **n (%)** |
| --- | --- | --- |
| **Ethnicity** | Jews | 708 (84.9) |
|  | Arabs | 126 (15.1) |
| **Gender** | Men | 411 (49.3) |
|  | Women | 423 (50.7) |
| **Age** | 18-24 | 136 (16.3) |
|  | 25-34 | 172 (20.6) |
|  | 35-44 | 160 (19.2) |
|  | 45-54 | 171 (20.5) |
|  | 55-64 | 108 (12.9) |
|  | 65+ | 88 (10.5) |
| **Earthquake risk in residential area** | High risk for earthquakes | 95 (11.4) |
|  | Low risk for earthquakes | 739 (88.6) |
| **Apartment ownership** | My apartment is owned by me or my parents | 558 (66.9) |
|  | My apartment is owned by relatives | 48 (5.7) |
|  | My apartment is rented/public housing/key money | 203 (24.3) |
|  | n/a | 26 (3.1) |
